# PhpR, as a unique transcriptional regulator, represses the expression of pyridoxal 5′-phosphate-binding protein YggS in *Alcaligenes*

**DOI:** 10.1128/aem.01399-26

**Published:** 2026-09-28

**Authors:** Qimiao Xu, Changchang Wang, Jiaxi Liu, Fuyu Song, De-Feng Li, Jiasong Li, Shang Dai, Xin Yan, Qing Hong, Jiandong Jiang, Shuang-Jiang Liu, Jian He, Jiguo Qiu

**Affiliations:** 1 Key Laboratory of Agricultural and Environmental Microbiology, Ministry of Agriculture and Rural Affairs, College of Life Sciences, Nanjing Agricultural University 98430, Nanjing, China; 2 State Key Laboratory of Microbial Resources, Environmental Microbiology Research Center at Institute of Microbiology, Chinese Academy of Sciences 85387, Beijing, China; 3 State Key Laboratory of Microbial Technology, Shandong University 520252, Qingdao, China; The University of Tennessee Knoxville, Knoxville, Tennessee, USA

**Keywords:** YggS, pyridoxal 5′-phosphate, MocR-family regulator PhpR, *Alcaligenes*

## Abstract

YggS (COG0325 family) was widely conserved across the three domains of life and had been implicated in pyridoxal 5′-phosphate (PLP) homeostasis. However, transcriptional regulators that directly controlled *yggS* had remained unknown. Here, using *Alcaligenes faecalis* JQ135, PhpR, a previously unrecognized MocR-family regulator, was identified as a transcriptional regulator that directly controlled *yggS*. Deletion of *yggS* in strain JQ135 impaired cell growth and caused intracellular PLP imbalance. PhpR (PLP homeostasis-related protein regulator, *phpR*) directly repressed *yggS* transcription by binding a conserved non-palindromic promoter motif in a PLP-dependent manner. Comparative genomic analyses further revealed that the adjacent *yggS-phpR* gene pair was divergently transcribed and highly conserved, but restricted to *Alcaligenes* species. Together, these findings established the first direct transcriptional regulatory mechanism for a YggS-family protein and revealed a PLP-responsive regulatory circuit linking cofactor homeostasis in bacteria.

**IMPORTANCE:**

Pyridoxal 5′-phosphate (PLP), the main catalytically active form of vitamin B6, was an essential cofactor for many enzymes, particularly those involved in amino acid metabolism. PLP deficiency could impair the activity of PLP-dependent enzymes and disrupt cellular metabolism, whereas excessive accumulation of free PLP could also cause metabolic imbalance. YggS-family proteins were widely conserved PLP homeostasis factors, yet how their expression was regulated had remained largely unknown. Here, we identified PhpR as a previously uncharacterized MocR-family transcriptional regulator that directly repressed the expression of YggS in *Alcaligenes*, establishing a direct link between intracellular PLP availability and *yggS* expression. These findings revealed a previously unknown regulatory mechanism governing bacterial PLP homeostasis and provided new insight into how PLP-responsive transcriptional regulation coordinated cofactor balance with cellular metabolism.

## INTRODUCTION

YggS (also known as PLPHP, PLPBP) was a highly conserved member of the COG0325 protein family that was distributed in all domains, including bacteria, plants, and animals (1–3). YggS family proteins were conserved pyridoxal 5′-phosphate (PLP)-binding proteins involved in PLP homeostasis. Although YggS adopted a fold related to type III PLP-dependent enzymes and bound PLP, it lacked the catalytic features of classical PLP enzymes and was therefore considered a noncatalytic PLP homeostasis factor rather than a metabolic catalyst (4, 5). Early genetic and metabolic studies established that loss of the *yggS* gene caused pleiotropic defects linked to disturbed vitamin B6 balance, including pyridoxine 5′-phosphate (PNP) accumulation, pyridoxine sensitivity, and widespread perturbation of amino acid (6) and one-carbon metabolism (7), thereby identifying YggS as a core determinant of intracellular PLP homeostasis (1, 8, 9). Subsequent work further suggested that YggS might contribute to balancing B6 vitamers by promoting the safe handling and turnover of protein-bound PLP, consistent with the need to maintain adequate cofactor supply while minimizing the toxicity of free PLP (10). Biochemical analyses later showed that *Escherichia coli* YggS bound PLP tightly through a solvent-exposed site and could transfer PLP to apo-serine hydroxymethyltransferase (SHMT/GlyA), supporting a role in cofactor delivery (5, 11). Together, these studies suggested that YggS contributed to PLP homeostasis and might participate in cofactor buffering or distribution, although the molecular basis underlying its physiological functions remained incompletely understood.

MocR-family transcriptional regulators represented a distinct subgroup of the GntR superfamily and typically consisted of an N-terminal winged helix-turn-helix (wHTH) DNA-binding domain and a C-terminal aminotransferase class-I/II (AAT-like) PLP-binding domain (12). MocR-family regulators have been implicated in diverse biological processes, including amino acid metabolism, vitamin biosynthesis, and stress responses (13), and the regulatory mechanisms and physiological ligands varied among different members (14). For example, GabR from *Bacillus subtilis* activated the *gabTD* operon for GABA catabolism in response to GABA (14, 15), whereas EhuR from *Sinorhizobium meliloti* repressed the *ehuABCD-eutABCDE* operon involved in ectoine uptake and catabolism, with DABA acting as its effector (16). Studies of PLP metabolism have identified two PLP-responsive MocR-family regulators, PdxR and PtsJ, which controlled canonical vitamin B6 biosynthetic pathways. PdxR activated *pdxST* transcription in the PLP biosynthetic pathway in *Bacillus clausii* (17), whereas PtsJ repressed *pdxK* in the salvage pathway in *Salmonella typhimurium* (18), indicating that PLP synthesis and recycling were both subject to direct transcriptional control by PLP-sensing regulators. However, these systems regulated the enzymes involved in PLP production or recycling, and no regulator directly controlling *yggS* itself had been identified to date. Despite substantial progress in defining the biochemical properties of YggS, how *yggS* was transcriptionally coordinated with cellular metabolic demand remained unknown.

In this study, we asked whether the YggS of *A. faecalis* JQ135 was subject to dedicated transcriptional regulation and, if so, what type of regulator mediated this control. We showed that deletion of *yggS* impaired bacterial growth and altered intracellular PLP levels, and further identified PhpR as a PLP-dependent MocR-family regulator that directly repressed *yggS* transcription. PhpR bound a conserved upstream motif of *yggS* and integrated PLP sensing with promoter recognition, thereby providing a mechanism for tuning *yggS* expression according to cellular cofactor status. Comparative genomic analyses further revealed that the divergently transcribed *yggS-phpR* module was conserved in *Alcaligenes*. These findings addressed a central unresolved question in YggS biology and provided a framework for understanding how PLP homeostasis was transcriptionally coordinated with bacterial growth and metabolism.

## RESULTS

### Role of *yggS* in regulating cellular growth

**Fig 1:**
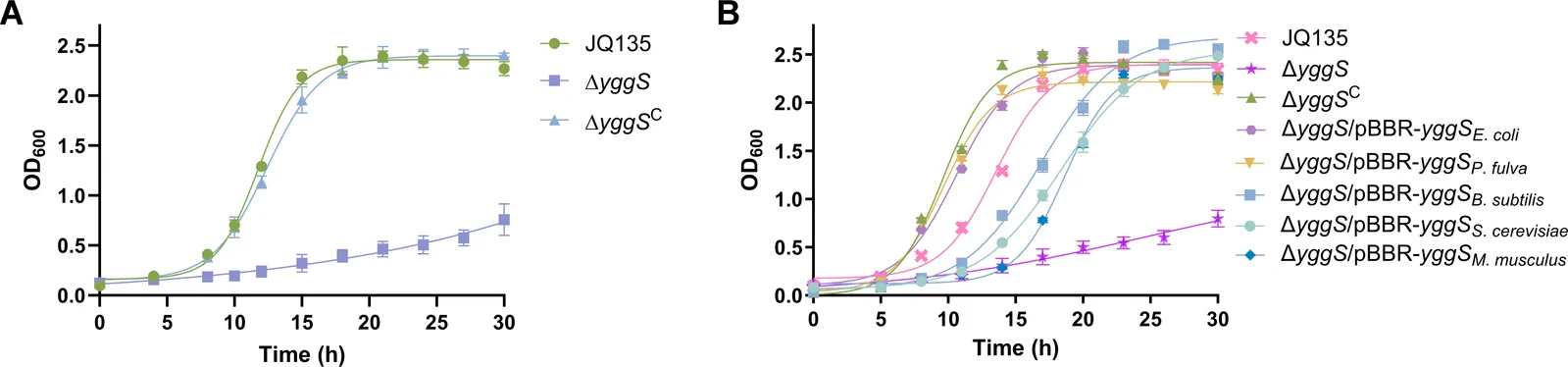
Role of YggS in growth in *A. faecalis* JQ135. (**A**) Growth of JQ135, Δ*yggS*, and the complemented strain (Δ*yggS*^C^) in HNM medium. (**B**) Effects of heterologous *yggS* homologs on growth in the Δ*yggS. yggS_E_*_. *coli*_: the *yggS* from *Escherichia coli* K-12, *yggS_P_*_. *fulva*_: the *yggS* from *Pseudomonas fulva* DSM17717, *yggS_B_*_. *subtilis*_: the *yggS* from *Bacillus subtilis* 168, *yggS_S_*_. *cerevisiae*_: the *yggS* from *Saccharomyces cerevisiae* BY4741, and *yggS_M_*_*. musculus*_: the *yggS* from *Mus musculus*.

To identify genes required for growth in *Alcaligenes faecalis* strain JQ135, a transposon mutagenesis library was constructed and screened. One mutant with limited growth was selected, and the transposon insertion was mapped to the *afa_08365* gene (GenBank: ASR91434.1), which encoded a YggS homolog (Fig. S1). To further confirm the physiological role of *yggS*, an in-frame, markerless deletion mutant Δ*yggS* was constructed (Fig. S2). Compared with the wild-type (WT) strain JQ135, the Δ*yggS* mutant exhibited markedly slower growth (Fig. 1A). Furthermore, the growth of the complemented strain Δ*yggS*^C^ was restored to WT strain level, confirming that the growth defect was attributable to deletion of *yggS*.

Given the important role of *yggS* in the normal growth of strain JQ135, we further investigated the phylogenetic distribution and sequence conservation of YggS homologs (PipY, PLPBP, PLPHP, etc.) across diverse taxa. A total of 6,719 representative amino acid sequences were retrieved using the KEGG Orthology identifier K06997. A phylogenetic tree was constructed (Fig. S3), and consistent with previous studies, members of this family were widely distributed across bacteria, archaea, and eukaryotes (3). The YggS protein from *A. faecalis* JQ135 clustered within the bacterial YggS/PLPHP family, supporting its classification as a conserved member of this protein family (Fig. S3). To assess whether YggS homologs from other species were functionally equivalent to YggS from *A. faecalis* JQ135, heterologous complementation assays were performed. Specifically, *yggS* homologs from *E. coli* K-12 (9), *Bacillus subtilis* 168, *Pseudomonas fulva* DSM17717, *Saccharomyces cerevisiae* BY4741, and *Mus musculus* were introduced into the Δ*yggS* mutant. As shown in Fig. 1B, complementation with these heterologous *yggS* homologs substantially alleviated the growth defect of the mutant. The genes from *P. fulva* and *E. coli* showed the strongest complementation effects, significantly increasing the growth rate and shortening the lag phase relative to the deletion strain (Fig. 1B).

### YggS, a PLP-binding protein, involved in intracellular PLP homeostasis

**Fig 2:**
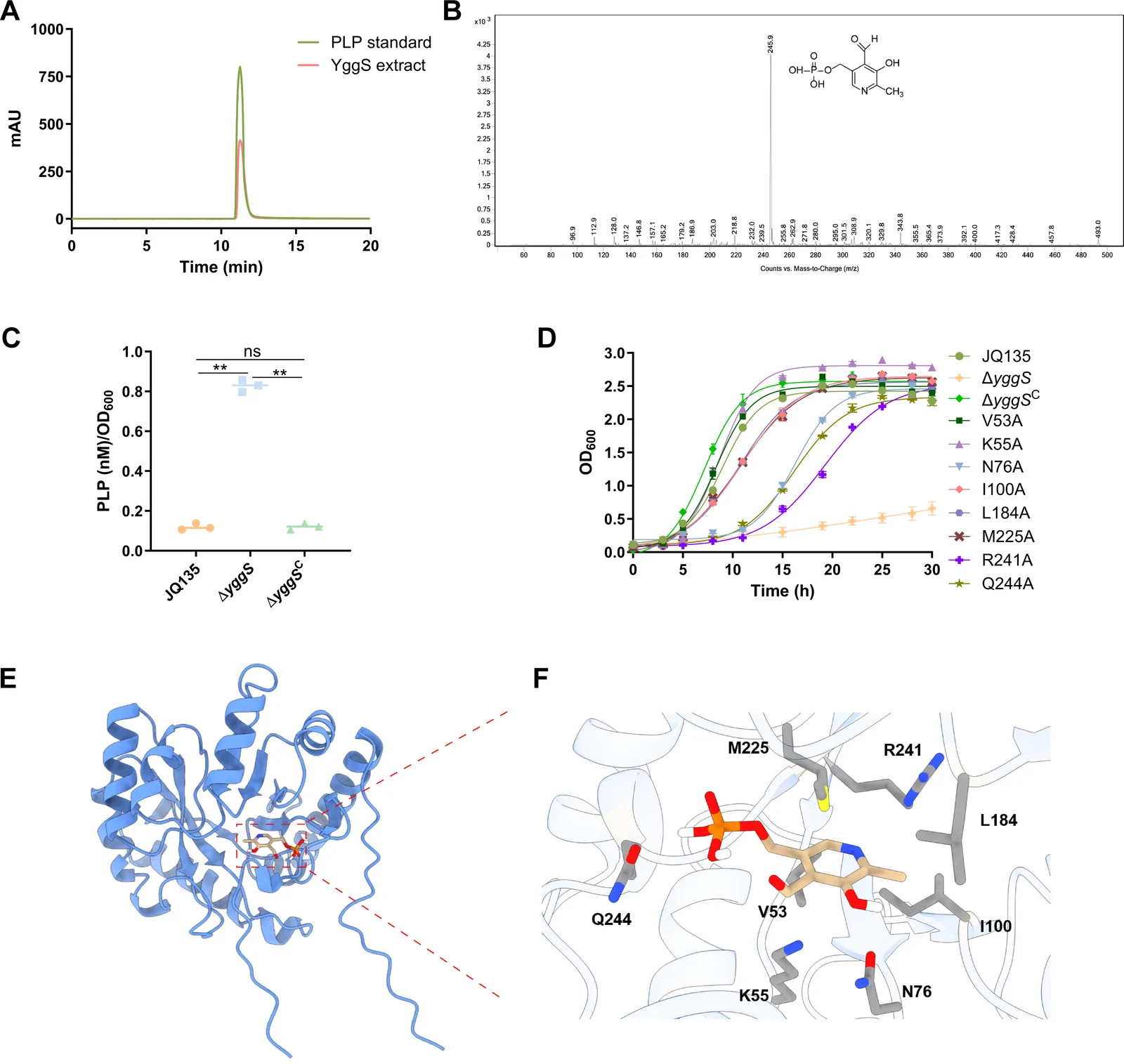
Structural and functional characterization of YggS and its association with PLP homeostasis. (**A**) HPLC analysis of recombinant YggS-bound cofactor with the PLP standard. (**B**) LC-MS spectrum confirming that the recombinant YggS-bound cofactor was PLP. (**C**) Intracellular PLP levels in WT, Δ*yggS*, and Δ*yggS*^C^. **, *P* < 0.01; ns, not significant. (**D**) Growth of *yggS* amino acid substitution mutants in *A*. *faecalis* JQ135. (**E**) Molecular docking analysis of YggS and PLP, visualized using PyMOL. (**F**) Close-up view of the PLP-binding pocket highlighting the key amino acid residues involved in PLP interactions.

Recombinant YggS from *A. faecalis* JQ135 was overexpressed and purified to determine whether it exhibited PLP-binding properties similar to those previously reported for YggS homologs (6, 8). The purified YggS was yellow, suggesting the presence of bound PLP. To release tightly bound cofactors, the purified protein was subjected to heat denaturation, and the resulting supernatant was analyzed by HPLC. As shown in Fig. 2A, the compound released from heat-treated YggS displayed the same retention time as the PLP standard. Then, the released compound was further analyzed by LC-MS. In negative ionization mode, a predominant ion with an *m/z* value of 245.9 was detected, which closely matched the expected molecular mass of PLP (Fig. 2B). Thus, the HPLC and LC-MS results suggested that YggS of *A. faecalis* was associated with PLP.

Structure-guided mutational analysis was performed to identify residues potentially associated with the predicted PLP-binding pocket (19). Based on three-dimensional structure prediction and molecular docking, eight residues (V53, K55, N76, I100, L184, M225, R241, and Q244) were located within or near the predicted PLP-binding pocket and were therefore selected for functional analysis (Fig. 2E and F; Fig. S4). These residues were individually substituted with alanine, and the resulting mutant alleles were introduced into the Δ*yggS* strain. Among them, N76A, R241A, and Q244A caused significant growth defects (Fig. 2D). At 15 h, the OD_600_ values of the N76A, R241A, and Q244A strains were 0.998, 0.652, and 0.933, respectively, all significantly lower than that of the WT strain (OD_600_ = 2.316; *P* < 0.001). In contrast, substitutions at the other predicted sites (V53, K55, I100, L184, and M225) had little effect on growth. Interestingly, previous studies identified the conserved lysine (K36 in *E. coli* YggS) as a key PLP-binding residue, with PLP covalently attached to K36 through a Schiff-base linkage (7). Subsequent studies showed that mutation of K36 of *E. coli* YggS alone did not completely abolish PLP binding (11). Consistent with this observation, mutation of the corresponding lysine residue (K55, Fig. S4) in YggS of *Alcaligenes* did not result in a significant growth defect (Fig. 2D), suggesting that other amino acid residues might partially compensate for the loss of lysine.

To determine whether disruption of *yggS* affected intracellular PLP homeostasis in *A. faecalis* JQ135, the WT strain, the Δ*yggS* mutant, and the complemented strain Δ*yggS*^C^ were grown in HNM medium (heterotrophic nitrification medium), and intracellular PLP levels were measured by HPLC after cell harvest and ultrasonication. Previous studies in *E. coli* and *Salmonella enterica* had shown that deletion of *yggS* primarily affected PLP distribution, particularly leading to extracellular PLP accumulation, while intracellular PLP levels were less affected. In contrast, the Δ*yggS* mutant of *A. faecalis* JQ135 exhibited a substantial increase in intracellular PLP levels, with approximately a 7.5-fold elevation compared with the WT strain (Fig. 2C). Similar alterations in PLP-related metabolism had been reported in *S. enterica* and *E. coli yggS* mutants, suggesting that YggS-family proteins were involved in regulating PLP homeostasis across diverse bacterial species (6, 8).

### PhpR negatively regulates *yggS* expression in a PLP-dependent manner and contributes to growth in *A*. *faecalis* JQ135

A putative MocR-family transcriptional regulator gene *phpR* (*afa_08370* gene, GenBank: ASR89455.1) was located divergently adjacent to *yggS* in *A. faecalis* JQ135, suggesting a potential regulatory relationship between these two genes. To determine whether PhpR affected cell growth, a markerless *phpR* deletion mutant (Δ*phpR*) and a double mutant lacking both *yggS* and *phpR* (Δ*yggS*Δ*phpR*) were constructed.

**Fig 3:**
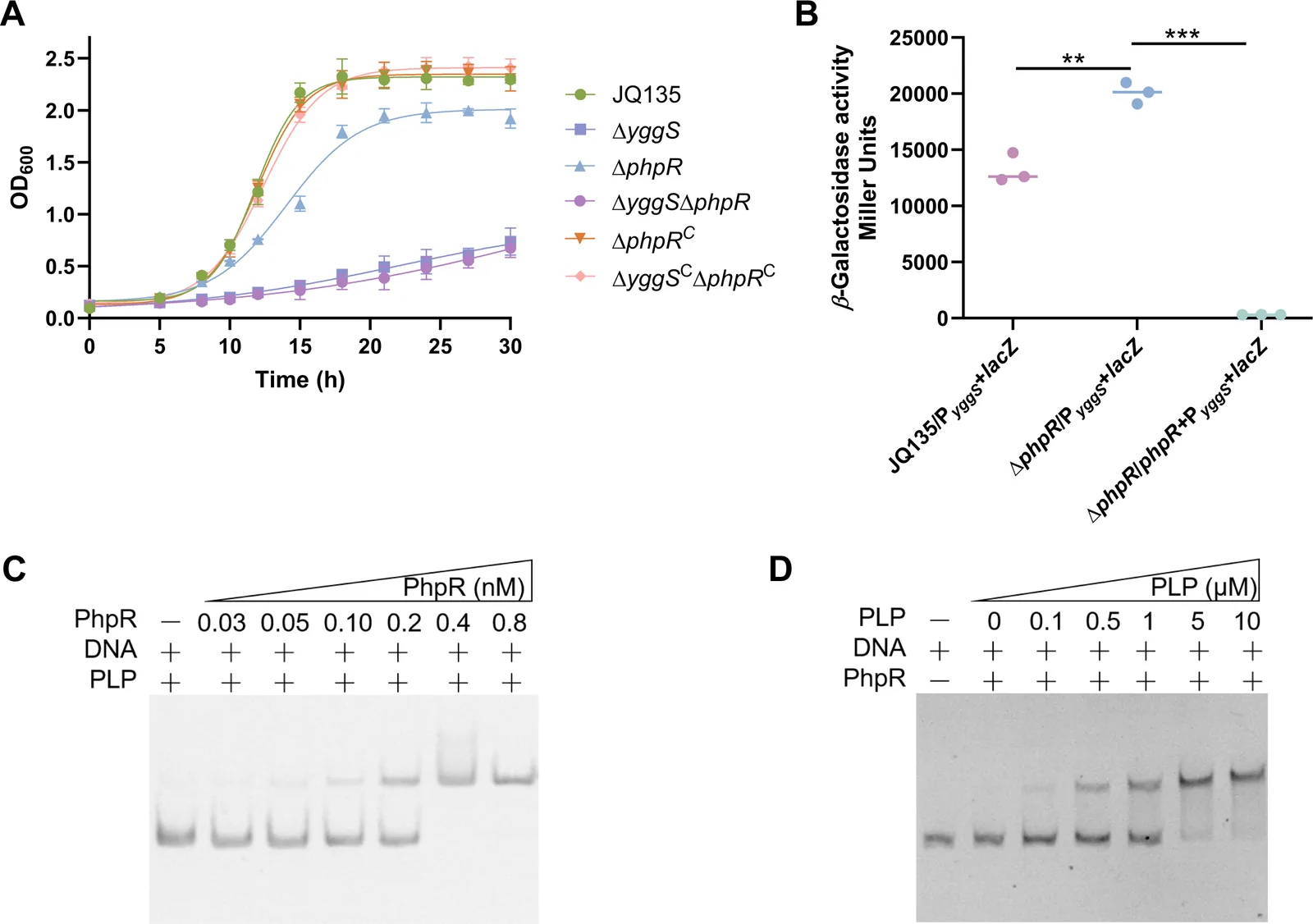
PhpR-mediated repression of *yggS* affects growth and promoter binding. (**A**) Growth curves of JQ135, Δ*yggS*, Δ*phpR*, the double mutant Δ*yggS*Δ*phpR*, and their corresponding complemented strains in HNM medium. (**B**) *β*-Galactosidase activity of the *yggS* promoter of JQ135/P*_yggS_-lacZ*, Δ*phpR*/P*_yggS_-lacZ*, and Δ*phpR*/*phpR* + P*_yggS_*-*lacZ*. Data are presented as mean ± SE (*n* = 3). **, *P* < 0.01 and ***, *P* < 0.001. (**C**) EMSAs of *yggS* promoter fragments with PLP and increasing purified PhpR. (**D**) EMSAs of *yggS* promoter fragments with purified PhpR (60 nM) and increasing PLP concentrations.

As shown in Fig. 3A, the Δ*yggS*Δ*phpR* double mutant exhibited a growth defect comparable to that of the Δ*yggS* mutant, with no significant difference between the two strains. At 15 h, the OD_600_ values of Δ*yggS* and Δ*yggS*Δ*phpR* were 0.321 and 0.266, respectively. By contrast, deletion of *phpR* alone caused a moderate reduction in growth. At 15 h, the cell density of the Δ*phpR* mutant (OD_600_ = 1.103) was significantly lower than that of JQ135 (OD_600_ = 2.185) (*P* < 0.01). The growth of complemented strains Δ*phpR*^C^ and Δ*yggS*^C^Δ*phpR*^C^ restored growth to wild-type levels. These results indicated that *yggS* was important for normal growth in *A. faecalis* JQ135 and that the growth defect caused by *phpR* deletion could be rescued by in *trans* expression of *phpR*.

To examine whether PhpR regulated *yggS* transcription, a *lacZ* reporter assay was performed. The *yggS* promoter was fused to *lacZ* in pHGEI03 to generate P*_yggS_-lacZ* (Fig. S5). As shown in Fig. 3B, compared to JQ135, the promoter activity of *yggS* in Δ*phpR* increased by 46%, whereas reintroduction of *phpR* into the Δ*phpR* (Δ*phpR/phpR +* P*_yggS_*-*lacZ*) caused a 61-fold reduction in reporter activity relative to Δ*phpR*. Together, these data showed that PhpR repressed *yggS* transcription. This result was further supported by electrophoretic mobility shift assay (EMSA). In the presence of 0.01 mM (10^4^ nM) PLP, a distinct *yggS* promoter DNA-protein complex was observed with 50 nM PhpR, and the shifted band became progressively stronger as the PhpR concentration increased, indicating direct and concentration-dependent binding of PhpR to the *yggS* promoter (Fig. 3C). Notably, 60 nM apo-PhpR alone did not produce a clear shifted band, whereas addition of 10 μM PLP resulted in a distinct DNA-protein complex with the *yggS* promoter. These results suggested that PLP acted as an effector molecule that enhanced the interaction between PhpR and the *yggS* promoter, thereby increasing the DNA-binding affinity of PhpR (Fig. 3D).

### Identification and conservation of the PhpR-binding motif in the *yggS* promoter

**Fig 4:**
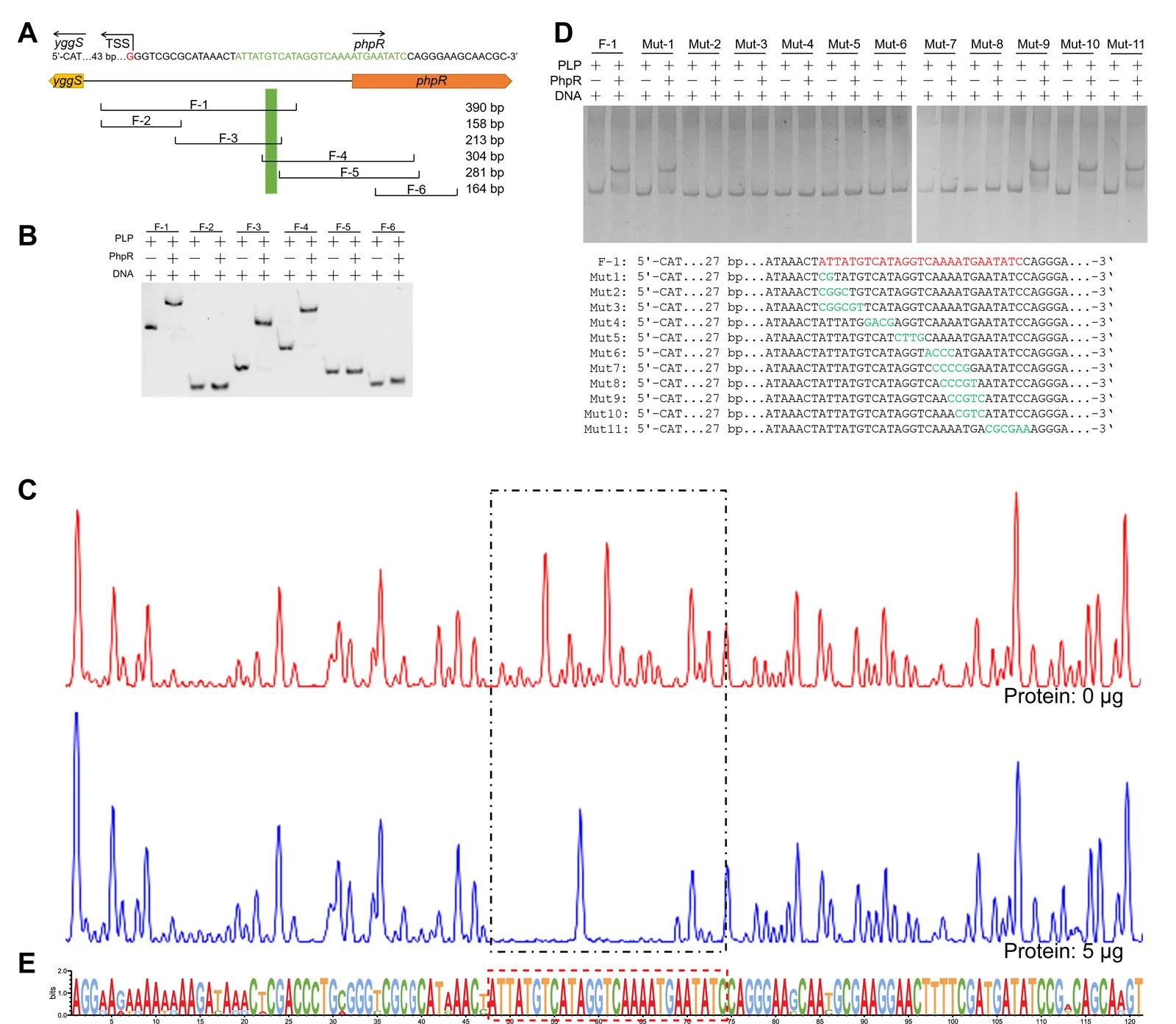
Identification and conservation of the PhpR-binding site in the *yggS* promoter. (**A**) Schematic diagram of the fragments F-1 to F-6. The nucleotide sequence and DNA elements of *yggS* promoter fragments are shown at the top. (**B**) EMSA of PhpR with the fragments F-1 to F-6. (**C**) The transcriptional start site (TSS) is indicated by arrowheads, and the *yggS* and *phpR* genes are shown by yellow and orange arrows, respectively. (**D**) EMSAs of the *yggS* promoter and its mutated variants (Mut-1 to Mut-11) with purified PhpR (100 nM). (**E**) Sequence logo representation of the conserved PhpR-binding motif identified in the genus *Alcaligenes*.

To delineate the PhpR-binding region within the *yggS* promoter, the transcription start site (TSS) of *yggS* was subsequently determined. Total RNA was extracted from *A. faecalis* JQ135 and subjected to TSS mapping. As shown in Fig. 4A, the TSS was located immediately upstream of the coding sequence, and the A residue of the ATG start codon was positioned at +44 relative to the mapped TSS.

Each fragment was independently incubated with purified PhpR protein and then analyzed by EMSA. As shown in Fig. 4B, clear mobility shifts were observed for fragments F-1, F-3, and F-4, whereas no obvious mobility shift was detected for fragments F-2, F-5, or F-6, indicating that PhpR specifically interacted with regions contained in F-1, F-3, and F-4. Notably, DNA-protein complexes were clearly detected with fragments F-1, F-3, and F-4, suggesting that the PhpR-binding site was located within their overlapping region. Consistent with this result, DNase I footprinting identified a single 27-bp protected region, 5′-GAT ATT CAT TTT GAC CTA TGA CAT AAT-3′, within the *yggS* promoter, indicating that this sequence represented the specific PhpR-binding site (Fig. 4C). No additional protected regions or repeated motifs were detected within the analyzed *yggS* promoter region, suggesting that PhpR recognized a single defined binding site in this promoter.

To further assess the functional importance of this binding site, a series of site-directed mutant promoter fragments, designated Mut-1 to Mut-11, was generated and examined by EMSA for PhpR binding. As shown in Fig. 4D, fragments Mut-1, Mut-9, Mut-10, and Mut-11 retained the ability to bind PhpR, whereas Mut-2, Mut-3, Mut-4, Mut-5, Mut-6, Mut-7, and Mut-8 failed to form PhpR-DNA complexes. These results indicated that the sequence 5′-TAT GTC ATA GGT CAA AAT G-3′ was essential for PhpR recognition and supported the conclusion that PhpR specifically bound a defined region within the *yggS* promoter.

Having identified the core sequence required for PhpR binding, we investigated whether the regulatory element was present in other members of *Alcaligenes*. To evaluate the conservation of this regulatory element, 112 *Alcaligenes* genome sequences were retrieved from the NCBI database and their upstream *yggS* promoter regions were compared. BLAST-based alignment showed that the overall nucleotide composition of the upstream regions varied among strains. In contrast, the PhpR-binding motif identified in *A. faecalis* JQ135 was completely conserved across all examined *Alcaligenes* genomes (Fig. 4E).

### Identification of key residues of PhpR

**Fig 5:**
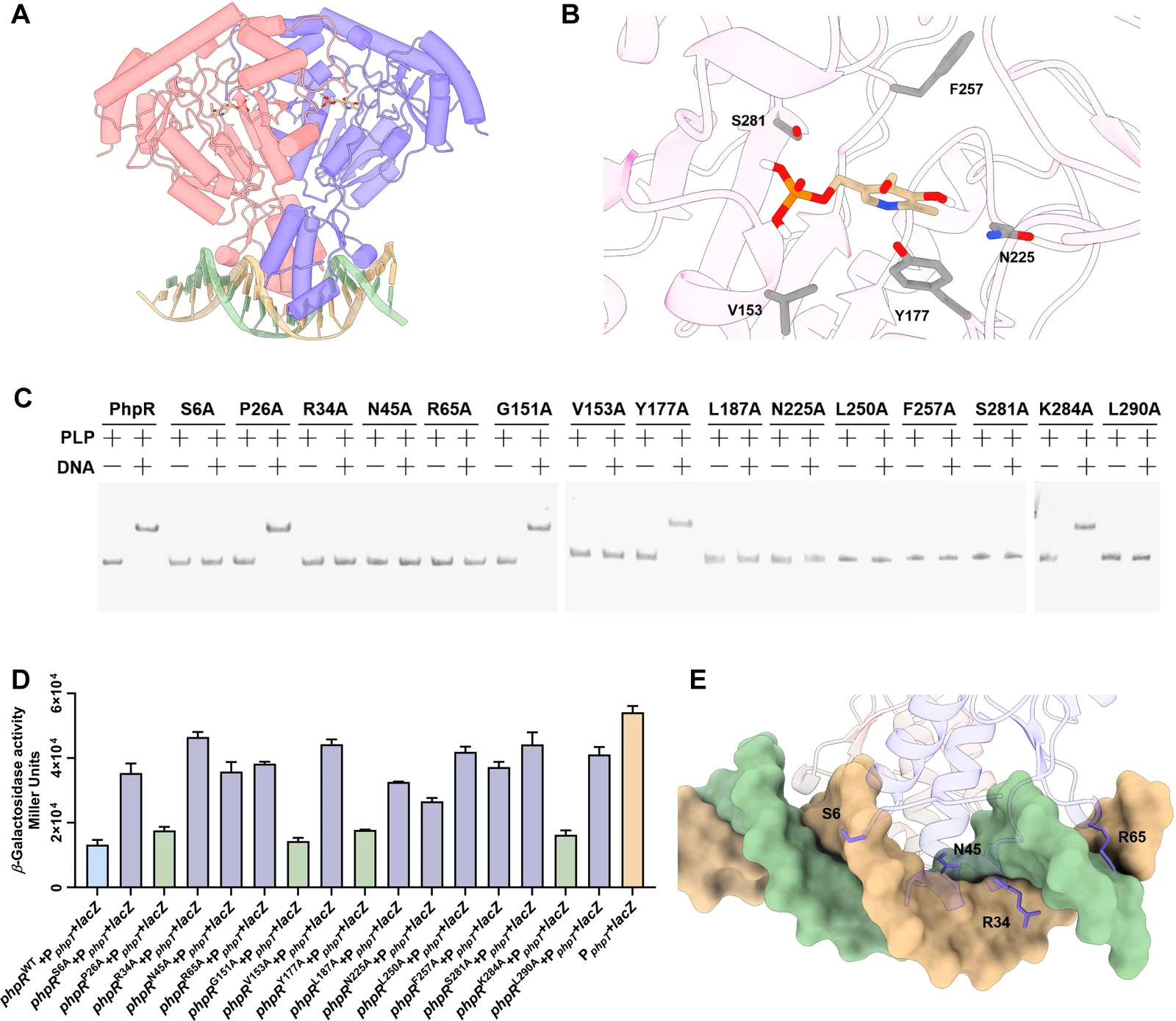
Identification of key residues of PhpR. (**A**) The analysis of molecular docking of PhpR with PLP and DNA was performed and visualized by Pymol. (**B**) Close-up view of the PhpR-PLP binding pocket and DNA interface, highlighting key amino acid residues. (**C**) EMSAs of the *yggS* promoter with purified PhpR and its key residue mutants (100 nM). (**D**) *β*-Galactosidase activity of the *yggS* promoter with purified PhpR and its key residue mutants. (**E**) Residues S6, R34, N45, and R65 of PhpR, predicted to participate in promoter recognition and DNA interaction, were highlighted as stick representations.

Several amino acid residues of PhpR were predicted to be involved in DNA binding and PLP interaction based on the predicted PhpR structure, NCBI CDD domain analysis, and molecular docking analysis (Fig. S6). Based on the predicted structure and PLP docking analysis, residues S6, P26, R34, N45, and R65 were located within the DNA-binding region, whereas residues G151, V153, Y177, L187, N225, L250, F257, S281, K284, and L290 were positioned around the predicted PLP-binding pocket (Fig. 5A and B; Fig. S6). To test these predictions, each candidate residue was individually substituted with alanine by site-directed mutagenesis, and the resulting PhpR variants were evaluated by both EMSA and *lacZ* reporter assays (Fig. 5C and D).

As shown in Fig. 5C, substitutions P26A, G151A, Y177A, and K284A did not detectably impair binding to the *yggS* promoter, as DNA-protein complexes comparable to those formed by the wild-type protein were retained. By contrast, substitutions at S6, R34, N45, R65, V153, L187, N225, L250, F257, S281, K284, and L290 abolished or markedly weakened promoter binding, indicating that these residues were important for PhpR-DNA interaction.

For the reporter assay, the *yggS* promoter was fused to *lacZ* in the shuttle plasmid pHGEI03 to generate P*_yggS_-lacZ*, whereas *phpR* or its mutant alleles were cloned into pBBR1MCS-5 and introduced together with the reporter construct into the indicated recipient strain by triparental mating (Fig. S7). Consistent with the EMSA results, the P26A, G151A, Y177A, and K284A variants displayed β-galactosidase activities comparable to that of wild-type PhpR (Fig. 5D), indicating that these substitutions did not measurably affect PhpR-mediated repression of *yggS* under the conditions tested. In contrast, variants defective in promoter binding showed elevated reporter activity to varying degrees, further supporting the conclusion that these residues were required for efficient repression of *yggS*.

Structural modeling and mutational analyses supported a mechanism for PhpR-mediated regulation. The predicted PhpR-DNA complex suggested that S6, R34, N45, and R65 participated in promoter recognition (Fig. 5E), whereas V153, L187, N225, L250, F257, S281, and L290 were predicted to contribute to PLP binding. Consistently, mutations affecting DNA-contact or PLP-binding residues impaired promoter binding and weakened PhpR-dependent repression, supporting a model in which PLP binding promoted a DNA-binding-competent conformation of PhpR.

To further examine the physiological importance of these residues, the corresponding amino acid substitution alleles were introduced into the Δ*phpR* background, and the resulting strains were assessed for growth. As shown in Fig. S8, substitutions P26A, G151A, Y177A, and K284A exhibited growth comparable to that of the strain complemented with wild-type *phpR*, indicating that these residues were dispensable for PhpR function under the conditions tested. In contrast, strains carrying substitutions S6A, R34A, N45A, R65A, V153A, L187A, N225A, L250A, F257A, S281A, or L290A displayed clear physiological defects, characterized by delayed growth to varying degrees (Fig. S8). Notably, these *in vivo* phenotypes closely paralleled the EMSA and *lacZ* reporter results, in which the same substitutions abolished or markedly weakened promoter binding and impaired repression of *yggS*. Together, these data indicated that the residues required for efficient binding of PhpR to the *yggS* promoter were also critical for its regulatory function *in vivo*, thereby linking promoter recognition directly to physiological output.

### A specific *yggS-phpR* regulatory module uniquely conserved in *Alcaligenes*

**Fig 6:**
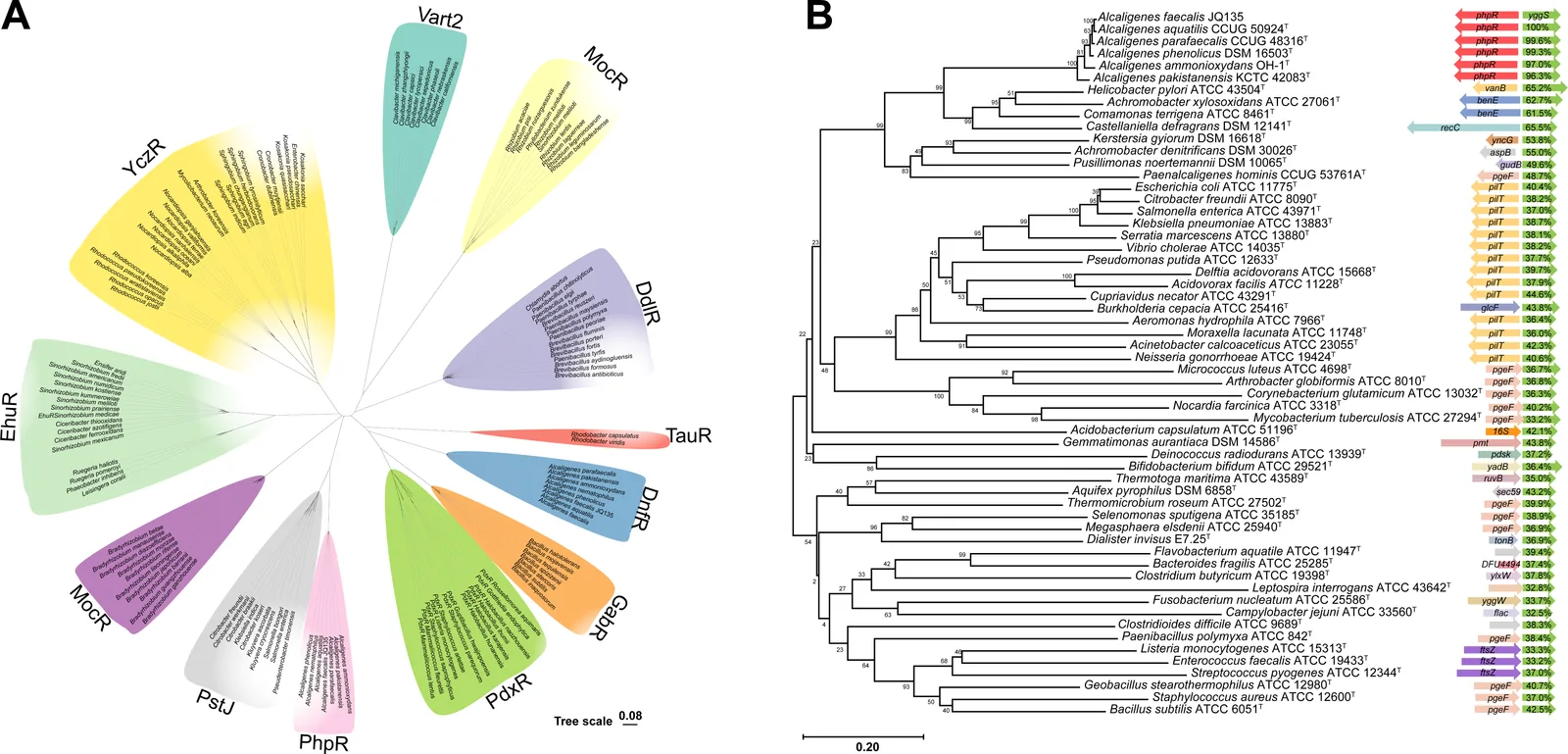
Phylogenetic distribution and genomic context of YggS and PhpR. (**A**) Phylogenetic classification showing taxonomic distribution of representative MocR family transcriptional regulators. (**B**) Phylogenetic tree and gene neighborhood analysis. In *Alcaligenes* species, *yggS* is consistently colocated with a divergently transcribed *phpR* regulator, whereas this arrangement is absent in other bacteria. The detailed gene names and protein accession numbers are listed in Table S4.

Bioinformatic analyses were performed to examine the evolutionary features of *phpR* and its target gene *yggS*. The *phpR* gene was found to be highly conserved within the genus *Alcaligenes*, with amino acid sequence identities generally exceeding 96% among homologs (Table S3). In contrast, the protein sequence of PhpR shared less than 50% amino acid sequence identity with previously characterized MocR-family transcriptional regulators. Consistent with this divergence, phylogenetic analysis showed that PhpR clustered in a distinct clade that was clearly separated from other reported MocR proteins, including EhuR, YczR, Vart2, MocR, DdlR, TauR, DnfR, GabR, PdxR, and PtsJ (Fig. 6A). Together with its low sequence similarity to previously characterized MocR regulators, this phylogenetic separation suggested that PhpR represented a previously unrecognized MocR-type transcriptional regulator in bacteria.

Given that PhpR directly regulated the transcription of *yggS*, the evolutionary conservation of YggS was further examined. Similar to PhpR, YggS was also highly conserved within *Alcaligenes*, with amino acid sequence identities exceeding 95% among homologs. By contrast, YggS displayed relatively limited identities to known YggS homologs from *E. coli*, *P. fulva*, *B. subtilis*, *S. cerevisiae*, and *M. musculus*, sharing 40.35%, 64.18%, 42.46%, 33.62%, and 37.50% sequence identities, respectively.

The strong conservation of both PhpR and YggS within *Alcaligenes* prompted further examination of whether this regulator-target module was maintained as a conserved genetic circuit. To address this, representative prokaryotic and eukaryotic species were selected for comparative analysis based on YggS phylogeny and local genomic organization. As shown in Fig. 6B, only *Alcaligenes* species exhibited the conserved co-localization of *yggS* with a divergently transcribed *phpR* gene. This arrangement was absent from all other examined bacteria, including closely related members of the order *Burkholderiales*, in which *yggS* was instead flanked by genes encoding unrelated proteins, such as BenE, PilT, or GlcF. Together, these findings identified the *yggS-phpR* circuit as a conserved genetic module restricted to the genus *Alcaligenes*, supporting the view that this regulatory module arrangement was genus-specific.

## DISCUSSION

In this study, we identified PhpR as a previously unrecognized MocR-family regulator that directly repressed the expression of YggS in *A. faecalis* JQ135. Deletion of *yggS* markedly impaired cellular growth, whereas *phpR* deletion resulted in a moderate growth defect. HPLC and LC-MS analyses further demonstrated that PLP was associated with YggS. Using a combination of the *lacZ* reporter assay, EMSA, and DNase I footprinting, we showed that PhpR bound a conserved non-palindromic motif in the *yggS* promoter in a PLP-dependent manner. Comparative genomic analyses further showed that the *yggS-phpR* organization and its associated motif were highly conserved but appeared to be restricted to *Alcaligenes* species, suggesting the evolution of a lineage-specific regulatory module for PLP homeostasis. Together, these findings established PhpR as the first experimentally validated transcriptional regulator directly controlling a *yggS* homolog.

PLP served as an essential cofactor for numerous metabolic enzymes but was also chemically reactive and potentially toxic when present in excess (10). This dual property necessitated tight regulation of intracellular PLP availability and balance. Previous studies established that YggS-family proteins contributed to PLP homeostasis by minimizing cofactor imbalance and protecting cellular metabolism from PLP-associated stress (20). Interestingly, the intracellular PLP accumulation observed in Δ*yggS* of *Alcaligenes* differed from previous reports in model organisms. In *E. coli* and *Salmonella*, YggS deficiency was mainly associated with extracellular PLP accumulation rather than a dramatic increase in intracellular PLP pools (6, 8). This distinct phenotype suggested that the role of YggS in PLP homeostasis might vary across bacterial species and metabolic contexts. The *A. faecalis* JQ135 and Δ*yggS* system therefore provided an additional model for investigating how YggS-mediated PLP homeostasis was coordinated with species-specific metabolic demands, as well as the physiological consequences and regulatory mechanisms of intracellular PLP imbalance.

In this context, PhpR represented a previously unrecognized regulatory layer within PLP homeostasis networks. Direct repression of *yggS* by PhpR was therefore likely to maintain YggS abundance within an optimal physiological range rather than permitting unrestricted expression. By fine-tuning the abundance of a PLP-binding protein according to cellular demand, the *yggS-phpR* module might help preserve metabolic balance, minimize the risk of PLP disequilibrium, and coordinate cofactor availability with cellular metabolism. Beyond its physiological significance, this regulatory strategy might have broader implications for microbial engineering and environmental biotechnology. Because PLP-dependent enzymes participate in numerous metabolic processes involved in carbon, nitrogen, sulfur, and amino acid metabolism, involving intracellular PLP homeostasis was essential for sustaining metabolic efficiency under changing environmental conditions. Regulatory modules such as PhpR-YggS provided a mechanism for fine-tuning cofactor availability without disrupting global metabolism, offering a potential strategy to improve the robustness, environmental adaptability, and catalytic performance of industrial and environmental microorganisms. More broadly, this work highlighted cofactor homeostasis as an underexplored target for engineering microbial metabolism, complementing conventional approaches that focused primarily on metabolic enzymes or regulatory pathways.

MocR-family transcriptional regulators exhibited diverse functions and participated in the regulation of various metabolic processes (14), with several members, including PtsJ and PdxR, being associated with PLP-related metabolism. Functionally, PhpR shared similarities with PtsJ of *Salmonella typhimurium* in that both proteins acted as PLP-responsive transcriptional repressors. However, the physiological targets of these regulators differed substantially. PtsJ repressed *pdxK*, which encoded pyridoxal kinase in the vitamin B6 salvage pathway, whereas PhpR directly regulated *yggS*, which encoded a conserved PLP-binding protein implicated in PLP homeostasis rather than PLP synthesis itself (18). PhpR also differed from PdxR of *Bacillus clausii*, which activated *pdxST* transcription while autorepressing its own gene, and in which PLP functioned as an anti-activator that reshaped promoter selectivity (17). Thus, whereas previously characterized MocR-family regulators primarily controlled PLP biosynthesis or salvage pathways, PhpR directly regulated the PLP-binding protein YggS. This finding therefore expanded the regulatory repertoire of MocR-family proteins and established a previously unrecognized regulatory model linking PLP sensing to control of intracellular PLP trafficking systems.

Our biochemical and genetic analyses further supported a model in which PhpR functioned as a PLP-responsive feedback regulator. Apo-PhpR displayed weak interaction with the *yggS* promoter, whereas PLP supplementation markedly enhanced promoter binding, suggesting that PLP promoted a DNA-binding-competent conformation of PhpR. This regulatory behavior was similar to GabR (15) and CRP (21), all of which required the presence of cofactors to bind to DNA. Together with the observed repression of *yggS* transcription, these results supported a negative-feedback mechanism, analogous to the classic *trp* repressor paradigm (22), in which elevated intracellular PLP levels stimulated PhpR-dependent repression of *yggS*, thereby linking intracellular PLP availability to the transcriptional regulation of this PLP-binding protein. Such a mechanism would allow dynamic adjustment of YggS abundance in response to metabolic demand and intracellular cofactor status.

The PhpR-binding site identified in *A. faecalis* JQ135 (5′-GAT ATT CAT TTT GAC CTA TGA CAT AAT-3′) (Fig. 4D) did not exhibit an obvious repeated motif commonly observed in some previously characterized MocR-family regulators (2). For example, GabR recognized an inverted-repeat-like motif (ATACCA-N_20–30_-ATACCA) (23, 24), whereas DnfR bound a direct-repeat element (GGTCTG-N_17_-GGTCTG) (25), indicating that many MocR-family regulators interacted with paired conserved motifs separated by variable spacers (14). By contrast, the motif identified here lacked an obvious perfect inverted repeat, exhibited only weak internal symmetry, and was highly conserved in the genus *Alcaligenes*. This atypical architecture may be consistent with a regulatory role in fine-tuning *yggS* expression rather than mediating a strict on/off transcriptional response. These observations therefore suggested that the PhpR-binding site represented a previously unrecognized MocR-type DNA-recognition motif and further expanded current understanding of the DNA-binding diversity and regulatory versatility of MocR-family transcription factors.

Members of the genus *Alcaligenes* were widely distributed across diverse natural and engineered environments, including soils, aquatic habitats, sediments, activated sludge, wastewater systems, and host-associated niches, reflecting substantial ecological breadth (26). Correspondingly, *Alcaligenes* exhibited remarkable metabolic versatility, including pollutant degradation (27), nitrogen transformation (28–30), biotransformation (31), antimicrobial activity (32), heavy-metal tolerance (33, 34), and carbon storage (35, 36). In this context, identification of the *yggS-phpR* module added a new dimension to the functional landscape of *Alcaligenes*. This study therefore expanded current understanding of both the biological significance of *Alcaligenes* and the broader principle that cofactor homeostasis could be directly coupled to cellular growth and metabolism.

## MATERIALS AND METHODS

### Bacterial strains, plasmids, and growth conditions

All bacterial strains and plasmids used in this study were listed in Table S1. *Alcaligenes faecalis* strain JQ135, a metabolically versatile bacterium capable of transforming nitrogen-containing compounds, was used as the model strain in this work (26, 37, 38). *A. faecalis* JQ135 was cultivated at 30°C in HNM medium containing (NH_4_)_2_SO_4_ (0.66 g/L), sodium succinate (4.72 g/L), KH_2_PO_4_ (0.5 g/L), NaH_2_PO_4_·12H_2_O (1.25 g/L), MgSO_4_·7H_2_O (0.2 g/L), NaCl (10 g/L), trace element solution (2.0 mL/L), and 5 M NaOH (100 mL). The trace element solution contained ZnSO_4_·7H_2_O (3.9 g/L), EDTA·2Na (57.1 g/L), CaCl_2_·2H_2_O (7.0 g/L), MnCl_2_·4H_2_O (1.0 g/L), FeSO_4_·7H_2_O (5.0 g/L), (NH4)_6_Mo7O_24_·4H_2_O (1.10 g/L), and CoCl_2_·6H_2_O (1.6 g/L), with the pH adjusted to 6.0 (39). *Escherichia coli* strains were routinely grown in LB medium at 37°C under standard laboratory conditions. When required, chloramphenicol (Cm, 30 μg/mL), gentamicin (Gm, 50 μg/mL), kanamycin (Km, 50 μg/mL), or streptomycin (Str, 50 μg/mL) was added to the medium for plasmid maintenance or selection of transformants. All cultures were handled according to standard microbiological procedures.

### Transposon mutagenesis and mapping of insertion sites

A transposon insertion library (40) was generated in *Alcaligenes faecalis* JQ135 using the transposon plasmid pSC123 (38). Mutants were selected on medium containing streptomycin and kanamycin. Genomic regions flanking the transposon insertion site were amplified by arbitrary PCR and sequenced. The resulting sequences were mapped to the JQ135 genome to identify the disrupted loci.

### Construction of mutants and genetic complementations

Gene deletions in *A. faecalis* JQ135 were generated using a two-step homologous recombination strategy with the suicide plasmid pJQ200SK (41, 42). Briefly, the upstream and downstream homologous arms were amplified using the primer pairs 5O/5I and 3I/3O, respectively, and the chloramphenicol resistance cassette (*Cm^r^*) was amplified with primers Cm-F/Cm-R. These fragments were fused by overlap-extension PCR through the complementary sequences introduced by primers 5I and 3I (43). The resulting fusion fragment was then cloned into pJQ200SK using the ClonExpress MultiS one-step cloning kit (Vazyme Biotech) and transformed into *E. coli* DH5α. After sequence verification, the resulting construct was subsequently introduced into *A. faecalis* JQ135. Single-crossover recombinants were selected on LB plates containing Str and Gm, whereas double-crossover mutants were screened on LB plates supplemented with Str, Cm, and 10% (wt/vol) sucrose. The desired deletion mutants were finally confirmed by DNA sequencing.

For genetic complementation, the broad-host-range plasmid pBBR1MCS-5 was used (44). The corresponding gene was amplified by PCR with gene-specific F/R primers, digested with the appropriate restriction enzymes, and ligated into pBBR1MCS-5 digested with the same enzymes. After transformation into *E. coli* DH5α and sequence verification, the resulting construct was introduced into the corresponding mutant for phenotypic analysis.

### Heterologous expression and purification of recombinant protein

Using His-tagged PhpR as an example, the target gene without its stop codon was amplified by PCR using primers F and R and cloned into the pET-29a(+) vector digested with *NdeI* and *XhoI*. The resulting recombinant plasmid was transformed into *E. coli* BL21(DE3) for protein expression. Recombinant strains were grown in LB medium at 37°C until the OD_600_ reached approximately 0.6, after which isopropyl *β*-d-1-thiogalactopyranoside (IPTG) was added to a final concentration of 0.1 mM. The cultures were then incubated at 16°C for 10 h to induce protein expression (45). Cells were harvested by centrifugation, and the recombinant 6 × His-tagged proteins were purified by Ni^2+^-nitrilotriacetic acid (Ni-NTA) agarose affinity chromatography (Vazyme Biotech). After elution with 200 mM imidazole, the proteins were dialyzed against 50 mM phosphate-buffered saline (Na_2_HPO_4_·12H_2_O, 15.46 g; NaH_2_PO_4_, 0.82 g; pH 7.4) at 4°C for 24 h. Protein purity was assessed by 12.5% SDS-PAGE.

PhpR variants were generated by site-directed mutagenesis using overlap-extension PCR with primers carrying the desired nucleotide substitutions (46). The mutated phpR coding sequences lacking the stop codon were amplified and cloned into the NdeI and XhoI sites of pET-29a(+) to generate C-terminal His-tagged constructs (47). All recombinant plasmids were verified by DNA sequencing and transformed into *Escherichia coli* BL21(DE3) for protein expression.

### EMSAs

All DNA fragments used for electrophoretic mobility shift assays (EMSAs) were amplified by PCR from the genomic DNA of *A. faecalis* JQ135 and purified using a DNA purification kit (Vazyme Biotech) (48). Purified PhpR protein was incubated with approximately 30 ng of each promoter probe in binding buffer containing 50 mM Tris-HCl (pH 7.0), 30 mM KCl, 5% (vol/vol) glycerol, 1 mM EDTA, and 5 mM DTT. The binding reactions were carried out at 25°C for 30 min. Protein-DNA complexes were then resolved on a 6% native polyacrylamide gel in 0.5 × Tris-glycine-EDTA buffer (49). After electrophoresis, the gels were stained with 4S Green Plus nucleic acid stain (Sangon Biotech, Shanghai, China), and the DNA bands were visualized under UV illumination.

To identify the key nucleotides within the PhpR-binding site (Fig. 4D), a series of point mutations were introduced by PCR. Using fragment Mut-1 as an example, two overlapping DNA subfragments were first amplified from the genomic DNA of *A. faecalis* JQ135 using the primer pairs Mut-1-F/EMSA-R and Mut-1-R/EMSA-F, respectively, thereby introducing the desired mutation. The two resulting subfragments were fused by overlap-extension PCR using the primers EMSA-F and EMSA-R to generate the full-length mutant Mut-1 DNA fragment containing the desired mutation (50). The resulting mutant fragments were purified and subsequently used as promoter probes for EMSA analysis.

### DNase I footprinting

The *yggS* promoter region (*P_yggS_*) and its truncated derivatives were amplified by PCR and cloned into the pMD19-T vector (TaKaRa). To generate fluorescently labeled probes for DNase I footprinting, the *P_yggS_* region was amplified from the recombinant pMD19-T-*P_yggS_* plasmid using 2 × TOLO HiFi DNA Polymerase Premix (TOLO Biotech, Shanghai, China) with primers M13F-47 and M13R-48, the latter of which was labeled at its 5′ end with 6-carboxyfluorescein (FAM) (25).

After purification using the Wizard SV Gel and PCR Clean-Up System (Promega, Madison, WI, USA), the FAM-labeled DNA probes were quantified using a NanoDrop 2000C spectrophotometer (Thermo Fisher Scientific, USA). DNase I footprinting assays were then performed as previously described. Briefly, 400 ng of the labeled probe was incubated with increasing concentrations of purified PhpR protein in a total volume of 40 μL at 25°C for 30 min (51). DNase I digestion was initiated by adding 10 μL of solution containing approximately 0.015 U of DNase I (Promega) and 100 nmol of freshly prepared CaCl_2_, followed by incubation at 37°C for 1 min. The reactions were terminated by addition of 140 μL DNase I stop solution consisting of 200 mM unbuffered sodium acetate, 30 mM EDTA, and 0.15% SDS. The samples were then extracted with phenol-chloroform, and the DNA was precipitated with ethanol. The resulting DNA pellets were resuspended in 30 μL of Milli-Q water. Preparation of the DNA ladder, electrophoresis conditions, and data analysis were carried out as previously described, except that the GeneScan LIZ600 size standard (Applied Biosystems) was used (52).

### *β*-Galactosidase assays

To evaluate the activity of the *yggS* promoter, a *P_yggS_-lacZ* transcriptional fusion plasmid was constructed. The *P_yggS_* fragment was amplified by PCR using the primers listed in Table S2. After digestion with *EcoRI* and *HindIII*, the PCR product was ligated into pHGEI03 digested with the same enzymes, yielding pHGEI03-*P_yggS_-lacZ* (53). The resulting recombinant plasmid was then introduced into *A. faecalis* JQ135 and the *phpR* deletion mutant to generate the strains JQ135/pHGEI03-*yggS*, Δ*phpR*/pHGEI03-*P_yggS_-lacZ*, and Δ*phpR*/pHGEI03-*phpR-P_yggS_-lacZ*.

*β*-Galactosidase activity was assayed as follows. Cells were harvested during the exponential growth phase by centrifugation, and 2 mL of culture was resuspended in 1 mL of Z-buffer (25, 54). The cells were permeabilized by addition of 50 μL of 0.1% (wt/vol) SDS and 100 μL of chloroform. *β*-Galactosidase activity was then measured using *o*-nitrophenyl-*β*-d-galactopyranoside (ONPG) as the substrate. Enzyme activity was expressed in Miller units and normalized to the OD_600_ and OD_420_ values (55). One Miller unit was defined as the amount of enzyme required to produce 1 μmol of *o*-nitrophenol (ONP) per minute. All assays were performed in triplicate (56).

### TSS of the *yggS* cluster

The transcription start site (TSS) of *yggS* was determined by 5′ rapid amplification of cDNA ends (5′ RACE) using the HiScript-TS 5′/3′ RACE Kit (Vazyme Biotech) according to the manufacturer’s instructions. Total RNA was isolated as described above and used as the template for RACE analysis. The final PCR products were purified using a gel extraction kit (Vazyme Biotech) and subsequently ligated into the pCE2 TA/Blunt-Zero vector using the Blunt-Zero Cloning Kit (Vazyme Biotech) (57). The resulting recombinant plasmids were then sequenced to identify the TSS.

### Analytical methods of PLP

PLP was quantified by HPLC using a Shimadzu AD20 system equipped with a Phecda C18 reversed-phase column (250 × 4.6 mm, 5 μm) and a photodiode array detector set at 380 nm. The mobile phase consisted of 0.10 mol/L KH_2_PO_4_ (pH 6.60), delivered at a flow rate of 1.0 mL/min, and the column temperature was maintained at 35°C (58). All experiments were performed in triplicate, and the results were presented as the mean ± SE.

LC-MS analysis was performed using an Agilent 1260 Infinity II liquid chromatography system coupled to an Agilent 6460 triple quadrupole mass spectrometer equipped with a Waters ACQUITY BEH C18 column (2.1 × 100 mm, 1.7 μm). The mobile phase consisted of acetonitrile and water containing 0.1% formic acid (95:5, vol/vol), with a flow rate of 0.2 mL/min, and the column temperature was maintained at 40°C. Mass spectrometric detection was carried out in negative electrospray ionization mode with full-scan acquisition over an *m/z* range of 50–500. The ion source temperature was set at 350°C, with nitrogen used as the nebulizing gas at 45 psi and 10 mL/min. The fragmentor voltage and capillary voltage were set to 80 and 350 V, respectively (59).

### Bioinformatic analysis and phylogenetic tree construction

Representative MocR-like transcription factors were selected based on previously reported classifications of functionally characterized MocR regulators (14), and their amino acid sequences were retrieved from the NCBI database. PhpR and the selected MocR homologs were aligned by multiple sequence alignment, and the resulting alignment was manually inspected before phylogenetic analysis (60). Phylogenetic trees were constructed in MEGA using the maximum-likelihood method with bootstrap analysis, and the resulting trees were visualized and annotated using Chiplot (61). Analyses of YggS homologs were performed using the same procedure.

### Structural prediction and molecular docking analysis

The three-dimensional structures of PhpR and YggS were obtained based on their amino acid sequences from UniProt. Conserved domains were analyzed using the NCBI Conserved Domain Database (CDD) to identify the predicted DNA-binding and AAT-like domains. Molecular docking analysis was performed to evaluate the potential interaction between PhpR and PLP, and residues located within the predicted binding pocket were selected as candidate PLP-interacting residues. The structural figures were generated using PyMOL (http://www.pymol.org) (62).

## Data Availability

The data that support the findings of this study are available from the corresponding author upon reasonable request.

## SUPPLEMENTAL MATERIAL

Supplemental material aem.01399-26-s0001.pdf Tables S1 to S4; Fig. S1 to S8.

ASM does not own the copyrights to Supplemental Material that may be linked to, or accessed through, an article. The authors have granted ASM a non-exclusive, world-wide license to publish the Supplemental Material files. Please contact the corresponding author directly for reuse.
