# Supplementary material for: PhpR, as a unique transcriptional regulator, represses the expression of pyridoxal 5′-phosphate-binding protein YggS in Alcaligenes
Source: Appl Environ Microbiol. 2026 Sep 28;92(10):e01399-26. doi: 10.1128/aem.01399-26 (PMC13689441; doi:10.1128/aem.01399-26)
Supplement: Supplementary file 1 [file aem.01399-26-s0001.pdf]

PhpR, as a unique transcriptional regulator, represses the expression of pyridoxal 5'-phosphate-binding protein YggS in *Alcaligenes*

Qimiao Xu[1], Changchang Wang[1], Jiaxi Liu[1], Fuyu Song[1], De-Feng Li[2], Jiasong Li[1], Shang Dai[1], Xin Yan[1], Qing Hong[1], Jiandong Jiang[1], Shuang-Jiang Liu[2,3], Jian He[1]*, Jiguo Qiu[1]*

[1] Key Laboratory of Agricultural and Environmental Microbiology, Ministry of Agriculture and Rural Affairs, College of Life Sciences, Nanjing Agricultural University, Nanjing, 210095, China;

[2] State Key Laboratory of Microbial Resources, and Environmental Microbiology Research Center at Institute of Microbiology, Chinese Academy of Sciences, Beijing, 100101, China;

[3] State Key Laboratory of Microbial Technology, Shandong University, Qingdao, 266273, China.

*Corresponding author:

Jiguo Qiu, Email: qiujiguo@njau.edu.cn; Tel: 86-25-84396685, Fax: 86-25-84396314

and

Jian He, Email: hejian@njau.edu.cn ; Tel: 86-25-84396314 , Fax: 86-25-84396314

Table S1 Strains and plasmids used in this study.

Strain or plasmid	Description	Reference or source
Strain		
A. faecalis JQ135	Str^r; wild type, versatile metabolism of nitrogen-containing compounds	CCTCC M 2015812
Mut-67	Str^r; Km^r; with a pSC123 transposon insertion in *yggS*	This study
JQ135Δ*phpR*	Str^r; Cm^r; *phpR* deletion mutant of JQ135	This study
JQ135Δ*yggS*	Str^r; Cm^r; *yggS* deletion mutant of JQ135	This study
JQ135Δ*yggS*Δ*phpR*	Str^r; Cm^r; *yggS* and *phpR* deletion mutant of JQ135	This study
JQ135Δ$yggS^{C}$	Str^r; Cm^r; Gm^r; JQ135Δ*yggS* containing pBBR1MCS-5-*yggS*	This study
JQ135Δ$phpR^{C}$	Str^r; Cm^r; Gm^r; JQ135Δ*phpR* containing pBBR1MCS-5-*phpR*	This study
JQ135Δ$yggS^{C}$Δ$phpR^{C}$	Str^r; Cm^r; Gm^r; JQ135Δ*phpR* containing pBBR1MCS-5-*phpR yggS*	This study
JQ135Δ*yggS*/pBBR1-$yggS_{E. coli}$	Str^r; Cm^r; Gm^r; JQ135Δ*yggS* containing pBBR1MCS-5-$yggS_{E. coli}$	This study
JQ135Δ*yggS*/pBBR1-$yggS_{P. fulva}$	Str^r; Cm^r; Gm^r; JQ135Δ*yggS* containing pBBR1MCS-5-$yggS_{P. fulva}$	This study
JQ135Δ*yggS*/pBBR1-$yggS_{B. subtilis}$	Str^r; Cm^r; Gm^r; JQ135Δ*yggS* containing pBBR1MCS-5-$yggS_{B. subtilis}$	This study
JQ135Δ*yggS*/pBBR1-$yggS_{S. cerevisiae}$	Str^r; Cm^r; Gm^r; JQ135Δ*yggS* containing pBBR1MCS-5-$yggS_{S. cerevisiae}$	This study
JQ135Δ*yggS*/pBBR1-$yggS_{M. musculus}$	Str^r; Cm^r; Gm^r; JQ135Δ*yggS* containing pBBR1MCS-5-$yggS_{M. musculus}$	This study
JQ135Δ*yggS*/pBBR1-$yggS^{V53A}$	Str^r, Gm^r; Cm^r; JQ135Δ*yggS* containing pBBR1MCS-5-$yggS^{V53A}$	This study
JQ135Δ*yggS*/pBBR1-$yggS^{K55A}$	Str^r, Gm^r; Cm^r; JQ135Δ*yggS* containing pBBR1MCS-5-$yggS^{K55A}$	This study
JQ135Δ*yggS*/pBBR1-$yggS^{N76A}$	Str^r; Cm^r; Gm^r; JQ135Δ*yggS* containing pBBR1MCS-5-$yggS^{N76A}$	This study
JQ135Δ*yggS*/pBBR1-$yggS^{I100A}$	Str^r; Cm^r; Gm^r; JQ135Δ*yggS* containing pBBR1MCS-5-$yggS^{I100A}$	This study
JQ135Δ*yggS*/pBBR1-$yggS^{L184A}$	Str^r; Cm^r; Gm^r; JQ135Δ*yggS* containing pBBR1MCS-5-$yggS^{L184A}$	This study
JQ135Δ*yggS*/pBBR1-$yggS^{M225A}$	Str^r; Cm^r; Gm^r; JQ135Δ*yggS* containing pBBR1MCS-5-$yggS^{M225A}$	This study
JQ135Δ*yggS*/pBBR1-$yggS^{R241A}$	Str^r; Cm^r; Gm^r; JQ135Δ*yggS* containing pBBR1MCS-5-$yggS^{R241A}$	This study
JQ135Δ*yggS*/pBBR1-$yggS^{Q244A}$	Str^r; Cm^r; Gm^r; JQ135Δ*yggS* containing pBBR1MCS-5-$yggS^{Q244A}$	This study
JQ135Δ*yggS*Δ*phpR*/pBBR1-$phpR^{S6A}$+P_{yggS}+*lacZ*	Str^r; Cm^r; Gm^r; Km^r; containing $phpR^{S6A}$ fragment inserted into pBBR1MCS-5 and the *yggS* pomoter inserted into pHGEI03	This study

JQ135Δ*yggS*Δ*phpR*/pBBR1-*phpR*^{P26A}+P$_{yggS}$+*lacZ*	Str^r; Cm^r; Gm^r; Km^r; containing *phpR*^{P26A} fragment inserted into pBBR1MCS-5 and the *yggS* pomoter inserted into pHGEI03	This study
JQ135Δ*yggS*Δ*phpR*/pBBR1-*phpR*^{R34A}+P$_{yggS}$+*lacZ*	Str^r; Cm^r; Gm^r; Km^r; containing *phpR*^{R34A} fragment inserted into pBBR1MCS-5 and the *yggS* pomoter inserted into pHGEI03	This study
JQ135Δ*yggS*Δ*phpR*/pBBR1-*phpR*^{N45A}+P$_{yggS}$+*lacZ*	Str^r; Cm^r; Gm^r; Km^r; containing *phpR*^{N45A} fragment inserted into pBBR1MCS-5 and the *yggS* pomoter inserted into pHGEI03	This study
JQ135Δ*yggS*Δ*phpR*/pBBR1-*phpR*^{R65A}+P$_{yggS}$+*lacZ*	Str^r; Cm^r; Gm^r; Km^r; containing *phpR*^{R65A} fragment inserted into pBBR1MCS-5 and the *yggS* pomoter inserted into pHGEI03	This study
JQ135Δ*yggS*Δ*phpR*/pBBR1-*phpR*^{G151A}+P$_{yggS}$+*lacZ*	Str^r; Cm^r; Gm^r; Km^r; containing *phpR*^{G151A} fragment inserted into pBBR1MCS-5 and the *yggS* pomoter inserted into pHGEI03	This study
JQ135Δ*yggS*Δ*phpR*/pBBR1-*phpR*^{Y177A}+P$_{yggS}$+*lacZ*	Str^r; Cm^r; Gm^r; Km^r; containing *phpR*^{Y177A} fragment inserted into pBBR1MCS-5 and the *yggS* pomoter inserted into pHGEI03	This study
JQ135Δ*yggS*Δ*phpR*/pBBR1-*phpR*^{L187A}+P$_{yggS}$+*lacZ*	Str^r; Cm^r; Gm^r; Km^r; containing *phpR*^{L187A} fragment inserted into pBBR1MCS-5 and the *yggS* pomoter inserted into pHGEI03	This study
JQ135Δ*yggS*Δ*phpR*/pBBR1-*phpR*^{N225A}+P$_{yggS}$+*lacZ*	Str^r; Cm^r; Gm^r; Km^r; containing *phpR*^{N225A} fragment inserted into pBBR1MCS-5 and the *yggS* pomoter inserted into pHGEI03	This study
JQ135Δ*yggS*Δ*phpR*/pBBR1-*phpR*^{L250A}+P$_{yggS}$+*lacZ*	Str^r; Cm^r; Gm^r; Km^r; containing *phpR*^{L250A} fragment inserted into pBBR1MCS-5 and the *yggS* pomoter inserted into pHGEI03	This study
JQ135Δ*yggS*Δ*phpR*/pBBR1-*phpR*^{F257A}+P$_{yggS}$+*lacZ*	Str^r, Gm^r; Cm^r; Km^r; containing *phpR*^{F257A} fragment inserted into pBBR1MCS-5 and the *yggS* pomoter inserted into pHGEI03	This study
JQ135Δ*yggS*Δ*phpR*/pBBR1-*phpR*^{S281A}+P$_{yggS}$+*lacZ*	Str^r; Cm^r; Gm^r; Km^r; containing *phpR*^{S281A} fragment inserted into pBBR1MCS-5 and the *yggS* pomoter inserted into pHGEI03	This study
JQ135Δ*yggS*Δ*phpR*/pBBR1-*phpR*^{K284A}+P$_{yggS}$+*lacZ*	Str^r; Cm^r; Gm^r; Km^r; containing *phpR*^{K284A} fragment inserted into pBBR1MCS-5 and the *yggS* pomoter inserted into pHGEI03	This study
JQ135Δ*yggS*Δ*phpR*/pBBR1-*phpR*^{L290A}+P$_{yggS}$+*lacZ*	Str^r; Cm^r; Gm^r; Km^r; containing *phpR*^{L290A} fragment inserted into pBBR1MCS-5 and the *yggS* pomoter inserted into pHGEI03	This study

JQ135/P_{yggS}+*lacZ*	Str^r; Km^r; For measuring *yggS* promoter activity in the presence of PhpR	This study
Δ*yggS*/P_{yggS}+*lacZ*	Str^r; Cm^r; Km^r; For measuring *yggS* promoter activity	This study
Δ*yggS*/*phpR*+P_{yggS}+*lacZ*	Str^r; Cm^r; Km^r; For measuring *yggS* promoter activity in the presence of PhpR	This study
Escherichia coli		
DH5*α*	Cloning host	Laboratory stock
HB101(pRK2013)	Help strain for triparental mating	Laboratory stock
HB101(pRK600)	Help strain for triparental mating	Laboratory stock
BL21(DE3)	F⁻ *ompT hsdS*(rB⁻ mB⁻) *gal dcm lacY1*(DE3)	Laboratory stock
Plasmids		
pET29a(+)	Km^r; expression plasmid	Laboratory stock
pJQ200SK	Gm^r; mob^+, *orip*15A, *lacZa*$^+$, *sacB*, suicide plasmid	[25]
pBBR1MCS-5	Gm^r; broad-host-range cloning plasmid	Laboratory stock
pSC123	Km^r; *sacB*-based suicide vector for homologous recombination	Laboratory stock
pHGEI03	Km^r; *lacZ* reporter plasmid	[53]
pCE2 TA/Blunt-Zero	Cloning plasmid for TSS	Vazyme Biotech
pET29a(+)-*yggS*	Km^r; containing *yggS* fragment inserted into pET29a(+)	This study
pET29a(+)-*phpR*	Km^r; containing *phpR* fragment inserted into pET29a(+)	This study
pET29a(+)-*phpR*^{S6A}	Km^r; containing *phpR*^{S6A} fragment inserted into pET29a(+)	This study
pET29a(+)-*phpR*^{P26A}	Km^r; containing *phpR*^{P26A} fragment inserted into pET29a(+)	This study
pET29a(+)-*phpR*^{R34A}	Km^r; containing *phpR*^{R34A} fragment inserted into pET29a(+)	This study
pET29a(+)-*phpR*^{N45A}	Km^r; containing *phpR*^{N45A} fragment inserted into pET29a(+)	This study
pET29a(+)-*phpR*^{R65A}	Km^r; containing *phpR*^{R65A} fragment inserted into pET29a(+)	This study
pET29a(+)-*phpR*^{G151A}	Km^r; containing *phpR*^{G151A} fragment inserted into pET29a(+)	This study
pET29a(+)-*phpR*^{V153A}	Km^r; containing *phpR*^{V153A} fragment inserted into pET29a(+)	This study
pET29a(+)-*phpR*^{Y177A}	Km^r; containing *phpR*^{Y177A} fragment inserted into pET29a(+)	This study
pET29a(+)-*phpR*^{L187A}	Km^r; containing *phpR*^{L187A} fragment inserted into pET29a(+)	This study

pET29a(+)-*phpR*^{N225A}	Km^r; containing *phpR*^{N225A} fragment inserted into pET29a(+)	This study
pET29a(+)-*phpR*^{L250A}	Km^r; containing *phpR*^{L250} fragment inserted into pET29a(+)	This study
pET29a(+)-*phpR*^{F257A}	Km^r; containing *phpR*^{F257A} fragment inserted into pET29a(+)	This study
pET29a(+)-*phpR*^{S281A}	Km^r; containing *phpR*^{S281A} fragment inserted into pET29a(+)	This study
pET29a(+)-*phpR*^{K284A}	Km^r; containing *phpR*^{K284A} fragment inserted into pET29a(+)	This study
pET29a(+)-*phpR*^{L290A}	Km^r; containing *phpR*^{L290A} fragment inserted into pET29a(+)	This study
pJQ-*yggS*	Gm^r; *yggS* gene deletion plasmid based on pJQ200SK	This study
pJQ-*phpR*	Gm^r; *phpR* gene deletion plasmid based on pJQ200SK	This study
pJQ- *yggS phpR*	Gm^r; *yggS phpR* gene deletion plasmid based on pJQ200SK	This study
pBBR1-*yggS*	Gm^r; pBBR1MCS-5 harboring *yggS* gene	This study
pBBR1-*yggS*^{V53A}	Gm^r; pBBR1MCS-5 harboring *yggS*^{V53A} gene	This study
pBBR1-*yggS*^{K55A}	Gm^r; pBBR1MCS-5 harboring *yggS*^{K55A} gene	This study
pBBR1-*yggS*^{N76A}	Gm^r; pBBR1MCS-5 harboring *yggS*^{N76A} gene	This study
pBBR1-*yggS*^{I100A}	Gm^r; pBBR1MCS-5 harboring *yggS*^{I100A} gene	This study
pBBR1-*yggS*^{L184A}	Gm^r; pBBR1MCS-5 harboring *yggS*^{L184A} gene	This study
pBBR1-*yggS*^{M225A}	Gm^r; pBBR1MCS-5 harboring *yggS*^{M225A} gene	This study
pBBR1-*yggS*^{R241A}	Gm^r; pBBR1MCS-5 harboring *yggS*^{R241A} gene	This study
pBBR1-*yggS*^{Q244A}	Gm^r; pBBR1MCS-5 harboring *yggS*^{Q244A} gene	This study
pBBR1-*yggS*$_{S.\ cerevisiae}$	Gm^r; pBBR1MCS-5 harboring *yggS* gene of *S. cerevisiae* BY4741	This study
pBBR1-*yggS*$_{B.\ subtilis}$	Gm^r; pBBR1MCS-5 harboring *yggS* gene of *B. subtilis* BS168	This study
pBBR1-*yggS*$_{P.\ fulva}$	Gm^r; pBBR1MCS-5 harboring *yggS* gene of *P. fulva* DSM 17717	This study
pBBR1-*yggS*$_{M.\ musculus}$	Gm^r; pBBR1MCS-5 harboring *yggS* gene of *Mus musculus*	This study
pBBR1-*yggS*$_{E.\ coli}$	Gm^r; pBBR1MCS-5 harboring *yggS* gene of *E. coli* K-12	This study
pBBR1-*phpR*	Gm^r; pBBR1MCS-5 harboring *phpR* gene	This study
pBBR1-*yggS phpR*	Gm^r; pBBR1MCS-5 harboring *yggS* and *phpR* gene	This study
pBBR1-*phpR*^{S6A}	Gm^r; pBBR1MCS-5 harboring *phpR*^{S6A} gene	This study

pBBR1-*phpR*^{P26A}	Gm^{r}; pBBR1MCS-5 harboring *phpR*^{P26A} gene	This study
pBBR1-*phpR*^{R34A}	Gm^{r}; pBBR1MCS-5 harboring *phpR*^{R34A} gene	This study
pBBR1-*phpR*^{N45A}	Gm^{r}; pBBR1MCS-5 harboring *phpR*^{N45A} gene	This study
pBBR1-*phpR*^{R65A}	Gm^{r}; pBBR1MCS-5 harboring *phpR*^{R65A} gene	This study
pBBR1-*phpR*^{G151A}	Gm^{r}; pBBR1MCS-5 harboring *phpR*^{G151A} gene	This study
pBBR1-*phpR*^{V153A}	Gm^{r}; pBBR1MCS-5 harboring *phpR*^{V153A} gene	This study
pBBR1-*phpR*^{Y177A}	Gm^{r}; pBBR1MCS-5 harboring *phpR*^{Y177A} gene	This study
pBBR1-*phpR*^{L187A}	Gm^{r}; pBBR1MCS-5 harboring *phpR*^{L187A} gene	This study
pBBR1-*phpR*^{N225A}	Gm^{r}; pBBR1MCS-5 harboring *phpR*^{N225A} gene	This study
pBBR1-*phpR*^{L250A}	Gm^{r}; pBBR1MCS-5 harboring *phpR*^{L250A} gene	This study
pBBR1-*phpR*^{F257A}	Gm^{r}; pBBR1MCS-5 harboring *phpR*^{F257A} gene	This study
pBBR1-*phpR*^{S281A}	Gm^{r}; pBBR1MCS-5 harboring *phpR*^{S281A} gene	This study
pBBR1-*phpR*^{K284A}	Gm^{r}; pBBR1MCS-5 harboring *phpR*^{K284A} gene	This study
pBBR1-*phpR*^{L290A}	Gm^{r}; pBBR1MCS-5 harboring *phpR*^{L290A} gene	This study
P_{yggS}+*lacZ*	For measuring *yggS* promoter activity	This study
phpR+P_{yggS}+*lacZ*	For measuring *yggS* promoter activity in the presence of PhpR	This study

Table S2 Primers used in this study.

Primers	Sequence(5'-3')	Description
yggS-5O	agggaacaaaagctggagctcggctgtgattatgggtgctgt	To construct plasmid pJQ-*yggS*
yggS-5I	tcttacgtgccgatcaacgtctcatccattacgcctgatcctgc	To construct plasmid pJQ-*yggS*
yggS-3O	agcttgatatcgaattcctgcaggctatcgacctcaatacccact	To construct plasmid pJQ-*yggS*
yggS-3I	cagttattggtgcccttaaacgcataaactattatgtcataggtcaaaatgaat	To construct plasmid pJQ-*yggS*
cm-F	cgtttaagggcaccaataactg	To clone of the Cm^r
cm-R	tgagacgttgatcggcacgtaaga	To clone of the Cm^r
phpR-5O	agcttgatatcgaattcctgcagtcgcttgtgtgatcggcat	To construct plasmid pJQ-*phpR*
phpR-5I	cagttattggtgcccttaaacgttagcccaagtcctgcaagaa	To construct plasmid pJQ-*phpR*
phpR-3O	agggaacaaaagctggagctcggaggtgtttacttgcacgaag	To construct plasmid pJQ-*phpR*
phpR-3I	tcttacgtgccgatcaacgtctcattgctgtcggatatcatcgaaa	To construct plasmid pJQ-*phpR*
yggS phpR-5O	agcttgatatcgaattcctgcagtcgcttgtgtgatcggcat	To construct plasmid pJQ-*yggS phpR*
yggS phpR-5I	cagttattggtgcccttaaacgttagcccaagtcctgcaagaa	To construct plasmid pJQ-*yggS phpR*
yggS phpR-3O	tcttacgtgccgatcaacgtctcatccattacgcctgatcctgc	To construct plasmid pJQ-*yggS phpR*
yggS phpR-3I	agggaacaaaagctggagctcggctgtgattatgggtgctgt	To construct plasmid pJQ-*yggS phpR*
yggS-F	acgcgaattttaacaaaatattaacgcccacgacgtccttgtgcat	To construct plasmid pBBR1-*yggS*
yggS-R	caatttcacacaggaaacagcttcatgatgttgttgccgtatcg	To construct plasmid pBBR1-*yggS*
phpR-F	caatttcacacaggaaacagctgtggcactggggcgaatt	To construct plasmid pBBR1-*phpR*
phpR-R	acgcgaattttaacaaaatattaacgcctaatagcgttcttgcaggacttgg	To construct plasmid pBBR1-*phpR*
yggS phpR-F	caatttcacacaggaaacagcttcttgagaacgccgctttga	To construct plasmid pBBR1-*yggS phpR*
yggS phpR-R	acgcgaattttaacaaaatattaacgctttaacgatctggatgggcg	To construct plasmid pBBR1-*yggS phpR*
E. coli-F	attcatgatgttgttgccgtatcttattttttagagtaatcacgcgcac	To express the *yggS* of *Escherichia coli*
E. coli-R	ttcttccttactggtgcgccatgaacgatattgcgcataacc	To express the *yggS* of *Escherichia coli*
P. fulva-F	ttcttccttactggtgcgccatgaccgagtacgccgaaga	To express the *yggS* of *Pseudomonas fulva*
P. fulva-R	attcatgatgttgttgccgtatctcaatggctggtggccag	To express the *yggS* of *Pseudomonas fulva*

B. subtilis-F	attcatgatgttgttgccgtatctcattgctgtacaccccctg	To express the *yggS* of *Bacillus subtilis*
B. subtilis-R	ttcttccttactggtgcgccttgcgtgttgttgataatttacgac	To express the *yggS* of *Bacillus subtilis*
S. cerevisia-F	ttcttccttactggtgcgccatgtccactggtattacttatgatgaaga	To express the *yggS* of *Saccharomyces cerevisiae*
S. cerevisia -R	attcatgatgttgttgccgtatcctaaatgattctagcttcatttttggag	To express the *yggS* of *Saccharomyces cerevisiae*
M. musculus-F	ttcttccttactggtgcgccatgttgagaggtggcagca	To express the *yggS* of *Mus musculus*
M. musculus-R	attcatgatgttgttgccgtatctcagtgtccttgcaccagt	To express the *yggS* of *Mus musculus*
pET29a(+)-*yggS*-F	aaggagatatacatatgatgtccgaatccgcccctg	To construct plasmid pET-29a(+)-*yggS*
pET29a(+)-*yggS*-R	tggtggtggtgctcgagtgatgttgttgccgtatcgg	To construct plasmid pET-29a(+)-*yggS*
pET29a(+)-*phpR*-F	aaggagatatacatatgatgaatatccagggaagcaa	To construct plasmid pET-29a(+)-*phpR*
pET29a(+)-*phpR*-R	tgctcgagatagcgttcttgcaggacttggg	To construct plasmid pET-29a(+)-*phpR*
yggS^{V53A}-F	aatcccgaaggttttgctggcagtaagtaaacggacat	To introduce a point mutation at V53 of the YggS
yggS^{V53A}-R	atgtccgtttacttactgccagcaaaaccttcgggatt	To introduce a point mutation at V53 of the YggS
yggS^{K55A}-F	ggtcaatcccgaaggtggcgctgacagtaagtaaacg	To introduce a point mutation at K55 of the YggS
yggS^{K55A}-R	cgtttacttactgtcagcgccaccttcgggattgacc	To introduce a point mutation at K55 of the YggS
yggS^{N76A}-F	gcttcctgcaccttggcctcgcccaataagcgac	To introduce a point mutation at N76 of the YggS
yggS^{N76A}-R	gtcgcttattgggcgaggccaaggtgcaggaagc	To introduce a point mutation at N76 of the YggS
yggS^{I100A}-F	ttgctttgcaggtgtcccgcaacagcccattccaaat	To introduce a point mutation at I100 of the YggS
yggS^{I100A}-R	atttggaatgggctgttgcgggacacctgcaaagcaa	To introduce a point mutation at I100 of the YggS
yggS^{L184A}-F	tcggcgtccatcaaagccgcggtcatcagacctttg	To introduce a point mutation at L184 of the YggS
yggS^{L184A}-R	caaaggtctgatgaccgcggctttgatggacgccga	To introduce a point mutation at L184 of the YggS
yggS^{M225A}-F	acttcatagtcacccgacgcacccatggatagttca	To introduce a point mutation at M225 of the YggS
yggS^{M225A}-R	tgaactatccatgggtgcgtcgggtgactatgaagt	To introduce a point mutation at M225 of the YggS
yggS^{R241A}-F	aaatggcttgtccaacggccacaatggtggagcct	To introduce a point mutation at R241 of the YggS
yggS^{R241A}-R	aggctccaccattgtggccgttggacaagccattt	To introduce a point mutation at R241 of the YggS
yggS^{Q244A}-F	gtgcaccaaaaatggccgctccaacgcgcacaatg	To introduce a point mutation at Q244 of the YggS
yggS^{Q244A}-R	cattgtgcgcgttggagcggccatttttggtgcac	To introduce a point mutation at Q244 of the YggS

phpR^{S6A}-F	aaagttccttcgcgttggctccctggatattcat	To introduce a point mutation at S6 of the PhpR
phpR^{S6A}-R	atgaatatccagggagccaacgcgaaggaacttt	To introduce a point mutation at S6 of the PhpR
phpR^{P26A}-F	gcggcaaaatctggccggcagcaattcgccccagt	To introduce a point mutation at P26 of the PhpR
phpR^{P26A}-R	actggggcgaattgctgccggccagattttgccgc	To introduce a point mutation at P26 of the PhpR
phpR^{R34A}-F	tgtttgagccagctcggctaccggcggcaaaatc	To introduce a point mutation at R34 of the PhpR
phpR^{R34A}-R	gattttgccgccggtagccgagctggctcaaaca	To introduce a point mutation at R34 of the PhpR
phpR^{N45A}-F	tatgccagggctaccgtggcccggttcaatcccag	To introduce a point mutation at N45 of the PhpR
phpR^{N45A}-R	ctgggattgaaccgggccacggtagccctggcata	To introduce a point mutation at N45 of the PhpR
phpR^{R65A}-F	ggatccgtgtgccacgggctccttgtgcatcggc	To introduce a point mutation at R65 of the PhpR
phpR^{R65A}-R	gccgatgcacaaggagcccgtggcacacggatcc	To introduce a point mutation at R65 of the PhpR
phpR^{G151A}-F	tcgattgcatcgaccgccgcgtagctgacgtgataat	To introduce a point mutation at G151 of the PhpR
phpR^{G151A}-R	attatcacgtcagctacgcggcggtcgatgcaatcga	To introduce a point mutation at G151 of the PhpR
phpR^{V153A}-F	cgctcgattgcatcgggcgcaccgtagctgacgtgat	To introduce a point mutation at V153 of the PhpR
phpR^{V153A}-R	atcacgtcagctacggtgcgcccgatgcaatcgagcg	To introduce a point mutation at V153 of the PhpR
phpR^{Y177A}-F	cgagttgatgctgttcagcgcgccaggatcttcaatcc	To introduce a point mutation at Y177 of the PhpR
phpR^{Y177A}-R	ggattgaagatcctggcgcgctgaacagcatcaactcg	To introduce a point mutation at Y177 of the PhpR
phpR^{L187A}-F	ccactggcttgtagcccgccgtatgcaccgagttg	To introduce a point mutation at L187 of the PhpR
phpR^{L187A}-R	caactcggtgcatacggcgggctacaagccagtgg	To introduce a point mutation at L187 of the PhpR
phpR^{N225A}-F	gtgagcatcccgtgggggcgtgcgcacgtggtgtc	To introduce a point mutation at N225 of the PhpR
phpR^{N225A}-R	gacaccacgtgcgcacgcccccacgggatgctcac	To introduce a point mutation at N225 of the PhpR
phpR^{L250A}-F	aagtggtcgtcaatcatcaacgccaattcgggatactgct	To introduce a point mutation at L250 of the PhpR
phpR^{L250A}-R	agcagtatcccgaattggcgttgatgattgacgaccactt	To introduce a point mutation at L250 of the PhpR
phpR^{F257A}-F	gctgctggccagcatccaggcgtggtcgtcaatcatcaa	To introduce a point mutation at F257 of the PhpR
phpR^{F257A}-R	ttgatgattgacgaccacgcctggatgctggccagcagc	To introduce a point mutation at F257 of the PhpR
phpR^{S281A}-F	cccagacccttggacacggcacgaatcaaagccca	To introduce a point mutation at S281 of the PhpR
phpR^{S281A}-R	tgggctttgattcgtgccgtgtccaagggtctggg	To introduce a point mutation at S281 of the PhpR

phpR^{K284A}-F	aaatccggccccagacccgcggacactgaacgaat	To introduce a point mutation at K284 of the PhpR
phpR^{K284A}-R	attcgttcagtgtccgcgggtctggggccggattt	To introduce a point mutation at K284 of the PhpR
phpR^{L290A}-F	caccagagccacgcgcgcatccggccccagacc	To introduce a point mutation at L290 of the PhpR
phpR^{L290A}-R	ggtctggggccggatgcgcgcgtggctctggtg	To introduce a point mutation at L290 of the PhpR
EMSA-F (EMSA-1-F)	caggcagcatcaattctttgtt	EMSA
EMSA-R (EMSA-1-R)	ccacgacgtccttgtgcat	EMSA
EMSA-F-2	ccgcagggtcgagtttatctt	EMSA
EMSA-F-3	atatccagggaagcaacgc	EMSA
EMSA-F-4-F	gggtcgcgcataaactattatgt	EMSA
EMSA-F-4-R	atcctgcaaggccgagct	EMSA
EMSA-F-5-F	gatgatatccgacagcaagtgg	EMSA
EMSA-5-R	tgggtgggcgcaggcaaa	EMSA
EMSA-6-F	gatgcacaaggacgtcgtgg	EMSA
EMSA-6-R	gtttgaccgcgtaccccag	EMSA
EMSA-Mut-1-F	gtcgcgcataaactcgtatgtcataggtcaaaatgaata	EMSA
EMSA-Mut-1-R	tattcattttgacctatgacatacgagtttatgcgcgac	EMSA
EMSA-Mut-2-F	gtcgcgcataaactcggctgtcataggtcaaaatgaata	EMSA
EMSA-Mut-2-R	tattcattttgacctatgacagccgagtttatgcgcgac	EMSA
EMSA-Mut-3-F	gtcgcgcataaactcggcgttcataggtcaaaatgaata	EMSA
EMSA-Mut-3-R	tattcattttgacctatgaacgccgagtttatgcgcgac	EMSA
EMSA-Mut-4-F	ataaactattatggacgaggtcaaaatgaatatccag	EMSA
EMSA-Mut-4-R	ctggatattcattttgacctcgtccataatagtttat	EMSA
EMSA-Mut-5-F	aactattatgtcatcttgcaaaatgaatatccaggga	EMSA
EMSA-Mut-5-R	tccctggatattcattttgcaagatgacataatagtt	EMSA
EMSA-Mut-6-F	ttatgtcataggtacccatgaatatccagggaagcaacgc	EMSA
EMSA-Mut-6-R	gcgttgcttccctggatattcatgggtacctatgacataa	EMSA

EMSA-Mut-7-F	tgtcataggtcccccggaatatccagggaagcaacgcg	EMSA
EMSA-Mut-7-R	cgcgttgcttccctggatattccgggggacctatgaca	EMSA
EMSA-Mut-8-F	tgtcataggtcacccgtaatatccagggaagcaacgcg	EMSA
EMSA-Mut-8-R	cgcgttgcttccctggatattacgggtgacctatgaca	EMSA
EMSA-TB-9-F	tgtcataggtcaaccgtcatatccagggaagcaacgcg	EMSA
EMSA-Mut-9-R	cgcgttgcttccctggatatgacggttgacctatgaca	EMSA
EMSA-Mut-10-F	tgtcataggtcaaacgtcatatccagggaagcaacgcg	EMSA
EMSA-Mut-10-R	cgcgttgcttccctggatatgacgtttgacctatgaca	EMSA
EMSA-Mut-11-F	tcataggtcaaaatgacgcgaaagggaagcaacgcgaag	EMSA
EMSA-Mut-11-R	cttcgcgttgcttccctttcgcgtcattttgacctatga	EMSA
YggS-GSP	accggcctggtaggccaggcggatgc	5' RACE
YggS-NGSP	ggccaggcggatgcggtcaatcccga	5' RACE
pHGEI03-P_{yggS}-F	ggtgaattcggtacccggttgctgtcggatatcatcgaaaa	To construct plasmid pHGEI03-P_{yggS}
pHGEI03-P_{yggS}-R	ccaagcttgtaatcatggtcatggcgcaccagtaaggaaga	To construct plasmid pHGEI03-P_{yggS}
pHGEI03-*phpR*-P_{yggS}- F	ggtgaattcggtacccggctaatagcgttcttgcaggacttg	To construct plasmid pHGEI03-P_{yggS}-*phpR*
pHGEI03-*phpR*-P_{yggS}- R	ccaagcttgtaatcatggtcatggcgcaccagtaaggaaga	To construct plasmid pHGEI03-P_{yggS}-*phpR*

Table S3 Amino Acid Sequence Similarity of PhpR Proteins from *Alcaligenes*.

Bacteria containing PhpR homolog	Accession ID of PhpR homolog	Query Cover	Per. Ident	Acc. Len
Alcaligenes facials JQ135	WP_094196523.1	100%	100.00%	436
Alcaligenes faecalis ATCC8750^{T}	WP_045931477.1	100%	98.39%	436
Alcaligenes aquatilis CCUG 50996^{T}	WP_035267913.1	100%	98.39%	436
Alcaligenes phenolicus DSM 16503^{T}	WP_026482728.1	100%	98.17%	436
Alcaligenes parafaecalis DSM 13975^{T}	WP_207874254.1	100%	96.79%	436
Alcaligenes ammonioxydans OH-1^{T}	WP_003801184.1	100%	96.33%	436
Alcaligenes pakistanensis KCTC 42083^{T}	WP_189393013.1	100%	96.33%	436

Table S4 Gene names, functional annotations, strains, and protein accession numbers of the identified proteins in Fig. 6.

Strain	Gene	Annotation	Gene Accession ID	*yggS*	YggS Accession ID	YggS similarity with that of *A. faecalis* JQ135
Alcaligenes aquatilis	*phpR*	MocR transcriptional regulator	WP_094196523.1	*yggS*	WP_094198331.1	100%
Alcaligenes parafaecalis	*phpR*	MocR transcriptional regulator	WP_207874254.1	*yggS*	WP_153229967.1	99.6%
Alcaligenes phenolicus	*phpR*	MocR transcriptional regulator	WP_353639413.1	*yggS*	WP_009454526.1	99.3%
Alcaligenes ammonioxydans	*phpR*	MocR transcriptional regulator	WP_003801184.1	*yggS*	WP_003801186.1	97.0%
Alcaligenes pakistanensis	*phpR*	MocR transcriptional regulator	WP_189393013.1	*yggS*	WP_189393014.1	96.3%
Helicobacter pylori	*vanB*	PDR/VanB family oxidoreductase	WP_011088275.1	*yggS*	WP_003811326.1	65.2%
Achromobacter xylosoxidans	*benE*	Benzoate transport protein	WP_020928313.1	*yggS*	WP_201259124.1	62.7%
Comamonas terrigena	*benE*	Benzoate transport protein	WP_066534331.1	*yggS*	WP_312586721.1	61.5%
Castellaniella defragrans	*recC*	Exodeoxyribonuclease V gamma chain	WP_198408298.	*yggS*	WP_465056791.1	65.5%
Kerstersia gyiorum	*yncG*	glutathione S-transferase-like protein	WP_068371555.1	*yggS*	WP_130487548.	53.8%
Achromobacter denitrificans	*aspB*	Aspartate aminotransferase	WP_062684263.1	*yggS*	WP_062684264.1	55.0%
Pusillimonas noertemannii	*gudB*	putative acyl-CoA thioester hydrolase	WP_017524937.1	*yggS*	WP_116518385.1	49.6%
Paenalcaligenes hominis	*pgeF*	Multicopper polyphenol oxidase	WP_167661481.1	*yggS*	WP_371019363.1	48.7%
Escherichia coli	*pilT*	Twitching motility protein PilT	WP_001326494.1	*yggS*	WP_000997795.1	40.4%
Citrobacter freundii	*pilT*	Twitching motility protein PilT	WP_119174540.1	*yggS*	WP_038633606.1	38.2%
Salmonella enterica	*pilT*	Twitching motility protein PilT	WP_001055649.1	*yggS*	WP_001055649.1	37.0%

Klebsiella pneumoniae	*pilT*	Twitching motility protein PilT	WP_014343425.1	*yggS*	WP_004144780.1	38.7%
Serratia marcescens	*pilT*	Twitching motility protein PilT	WP_033649588.1	*yggS*	WP_033639392.1	38.1%
Vibrio cholerae	*pilT*	Twitching motility protein PilT	WP_000350195.1	*yggS*	WP_001256650.1	38.2%
Pseudomonas putida	*pilT*	Twitching motility protein PilT	WP_049588310.1	*yggS*	WP_010955637.1	37.7%
Delftia acidovorans	*pilT*	Twitching motility protein PilT	WP_012202999.1	*yggS*	WP_315529980.1	39.7%
Acidovorax facilis	*pilT*	Twitching motility protein PilT	WP_055398404.1	*yggS*	WP_426306120.1	37.9%
Cupriavidus necator	*pilT*	Twitching motility protein PilT	WP_010815050.1	*yggS*	WP_010815051.1	44.6%
Burkholderia cepacia	*glcF*	Glycolate dehydrogenase iron-sulfur subunit	WP_021163859.1	*yggS*	WP_153487861.1	43.8%
Aeromonas hydrophila	*pilT*	Twitching motility protein PilT	WP_011707389.1	*yggS*	WP_378008736.1	36.4%
Moraxella lacunata	*pilT*	Twitching motility protein PilT	WP_065256236.1	*yggS*	WP_062499975.1	36.0%
Acinetobacter calcoaceticus	*pilT*	Twitching motility protein PilT	WP_003653239.1	*yggS*	WP_005048579.1	42.3%
Neisseria gonorrhoeae	*pilT*	Twitching motility protein PilT	WP_003690200.1	*yggS*	WP_003690201.1	40.6%
Micrococcus luteus	*pgeF*	Multicopper polyphenol oxidase	WP_010078506.1	*yggS*	WP_115166147.1	36.7%
Arthrobacter globiformis	*pgeF*	Multicopper polyphenol oxidase	WP_003801744.1	*yggS*	WP_307101288.1	36.8%
Corynebacterium glutamicum	*pgeF*	Multicopper polyphenol oxidase	WP_003856519.1	*yggS*	WP_060564798.1	36.3%
Nocardia farcinica	*pgeF*	Multicopper polyphenol oxidase	WP_011208301.1	*yggS*	PFX05632.1	40.2%
Mycobacterium tuberculosis	*pgeF*	Multicopper polyphenol oxidase	NP_216665.1	*yggS*	AOT74140.1	33.2%
Acidobacterium capsulatum	*rsmE*	16S rRNA (uracil(1498)-N(3))-methyltransferase	WP_015897397.1	*yggS*	WP_015897396.1	42.1%
Gemmatimonas aurantiaca	*pmt*	putative membrane protein	WP_156798937.1	*yggS*	WP_083765519.1	43.8%

Deinococcus radiodurans	*pdsk*	Pyridoxal 5'-phosphate synthase	WP_027480030.1	*yggS*	WP_010888009.1	37.2%
Bifidobacterium bifidum	*yadB*	Glutamyl-Q tRNA(Asp) synthetase	WP_230958129.1	*yggS*	WP_033510141.1	36.4%
Thermotoga maritima	*ruvB*	Holliday junction ATP-dependent DNA helicase	WP_004082245.1	*yggS*	WP_004082246.1	35.0%
Aquifex pyrophilus	*Sec59*	Dolichol kinase	WP_459774954.1	*yggS*	WP_459774953.1	43.2%
Thermomicrobium roseum	*pgeF*	Multicopper polyphenol oxidase	WP_012642205.1	*yggS*	WP_505156842.1	39.9%
Selenomonas sputigena	*pgeF*	Multicopper polyphenol oxidase	WP_006192946.1	*yggS*	CAP6907648.1	38.9%
Megasphaera elsdenii	*pgeF*	Multicopper polyphenol oxidase	WP_014015162.1	*yggS*	WP_014015161.1	36.9%
Dialister invisus	*tonB*	TonB family protein	WP_417036602.1	*yggS*	WP_486844322.1	36.9%
Flavobacterium aquatile	hypothetical protein	hypothetical protein	WP_035128342.1	*yggS*	WP_035128340.1	39.4%
Bacteroides fragilis	*DFU4494*	hypothetical protein DUF4494	WP_005788684.1	*yggS*	WP_010993146.1	37.4%
Clostridium butyricum	*yxlW*	DUF881 domain-containing protein	WP_002580586.1	*yggS*	WP_455793946.1	37.8%
Leptospira interrogans	hypothetical protein	hypothetical protein	WP_001250524.1	*yggS*	WP_000538449.1	32.8%
Fusobacterium nucleatum	*yggW*	oxidase-like protein YggW	WP_011016482.1	*yggS*	WP_338938627.1	33.7%
Campylobacter jejuni	*flac*	flagellin C	WP_057096028.1	*yggS*	WP_002852054.1	32.5%
Clostridioides difficile	*gcvH*	hypothetical protein	WP_009903276.1	*yggS*	WLD28977.1	38.3%
Paenibacillus polymyxa	*pgeF*	Multicopper polyphenol oxidase	WP_026065490.1	*yggS*	WP_484695583.1	38.4%
Listeria monocytogenes	*ftsZ*	Cell division protein FtsZ	WP_003731982.1	*yggS*	WP_003726459.1	33.3%
Enterococcus faecalis	*ftsZ*	Cell division protein FtsZ	WP_002355899.1	*yggS*	WP_002358890.1	33.2%
Streptococcus pyogenes	*ftsZ*	Cell division protein FtsZ	WP_010922493.1	*yggS*	WP_010922493.1	37.0%
Geobacillus stearothermophilus	*pgeF*	Multicopper polyphenol oxidase	WP_033016140.1	*yggS*	WP_033011299.1	40.7%

Staphylococcus aureus	*pgeF*	Multicopper polyphenol oxidase	WP_000999341.1	*yggS*	WP_001548498.1	37.0%
Bacillus subtilis	*pgeF*	Multicopper polyphenol oxidase	WP_009967191.1	*yggS*	WP_088325833.1	42.5%

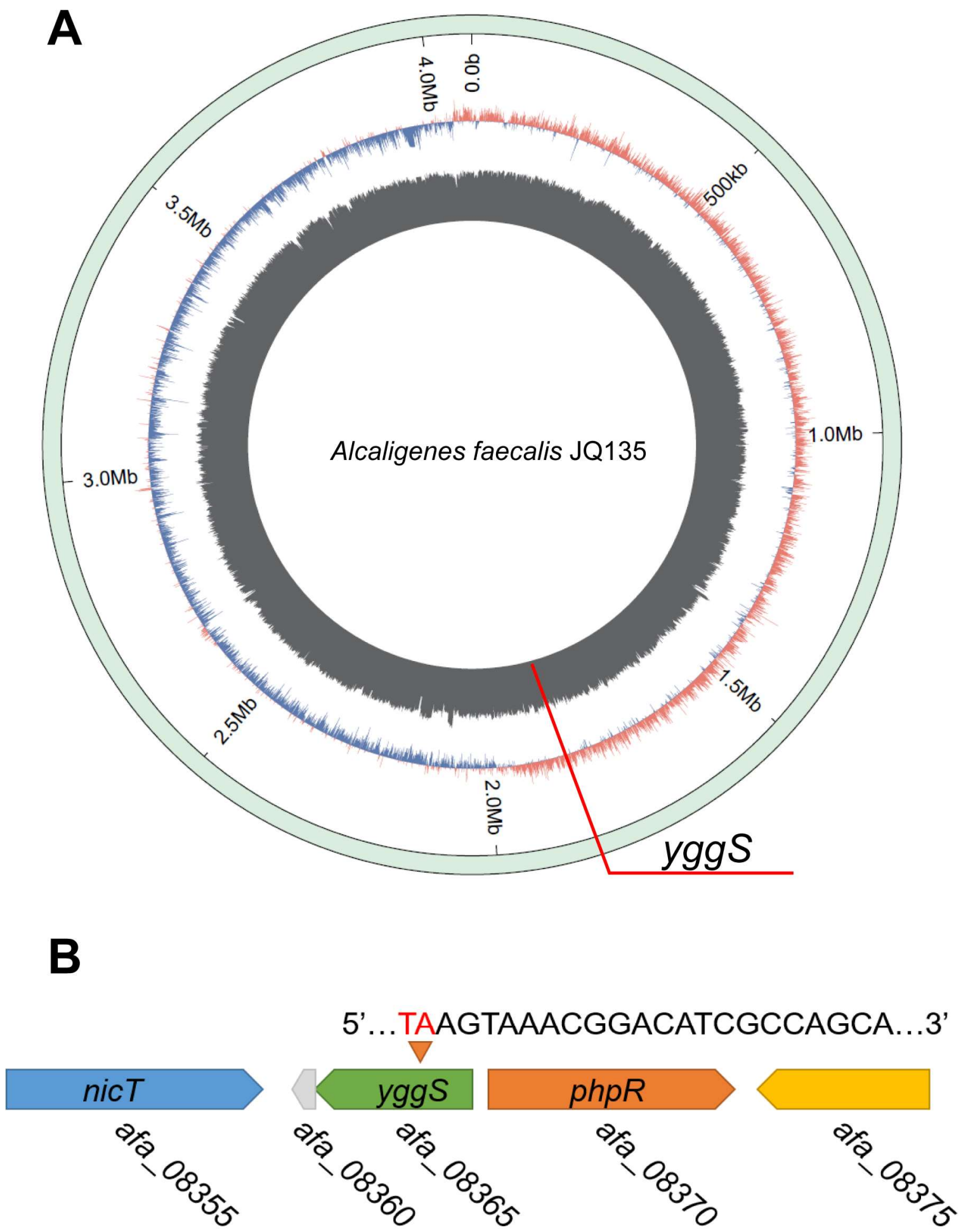

Figure S1 Schematic representation of the transposon insertion site in the mutant strain. (A) Circular genome map of *A. faecalis* JQ135 showing the chromosomal location of *yggS*. The red arrow indicates the position of *yggS* on the chromosome. (B) The organization and relative transcriptional orientations of the *yggS-phpR* locus are shown. The red sequence marks the transposon recognition site, indicating that the insertion is located within this region.

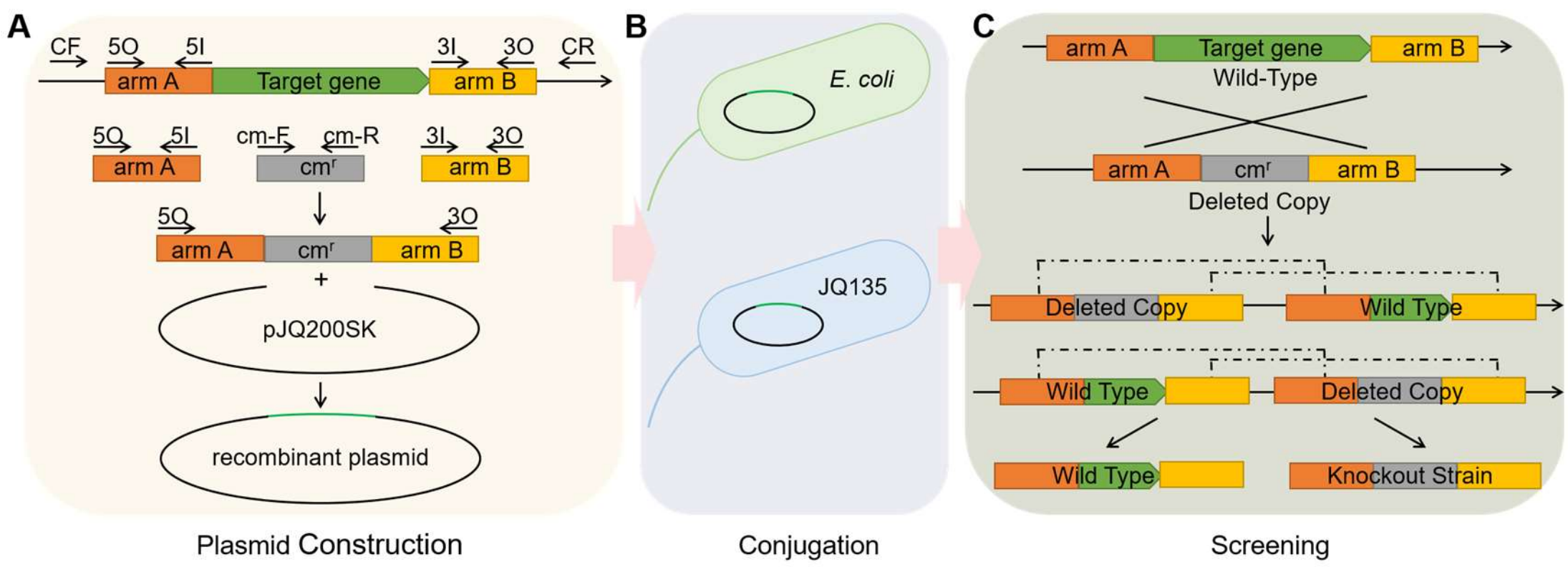

Figure S2 Schematic diagram of the construction and verification of the target gene deletion mutant in strain JQ135. (A) construction of the recombinant suicide plasmid pJQ200SK carrying the upstream (arm A) and downstream (arm B) homologous regions flanking the target gene. (B) conjugative transfer of the recombinant plasmid from *E. coli* donor cells into *A. faecalis* JQ135. (C) screening strategy for homologous recombination events. The first crossover generated a single-crossover intermediate containing both the wild-type and deleted copies, whereas the second crossover resulted in either restoration of the wild-type allele or generation of the target gene knockout mutant.

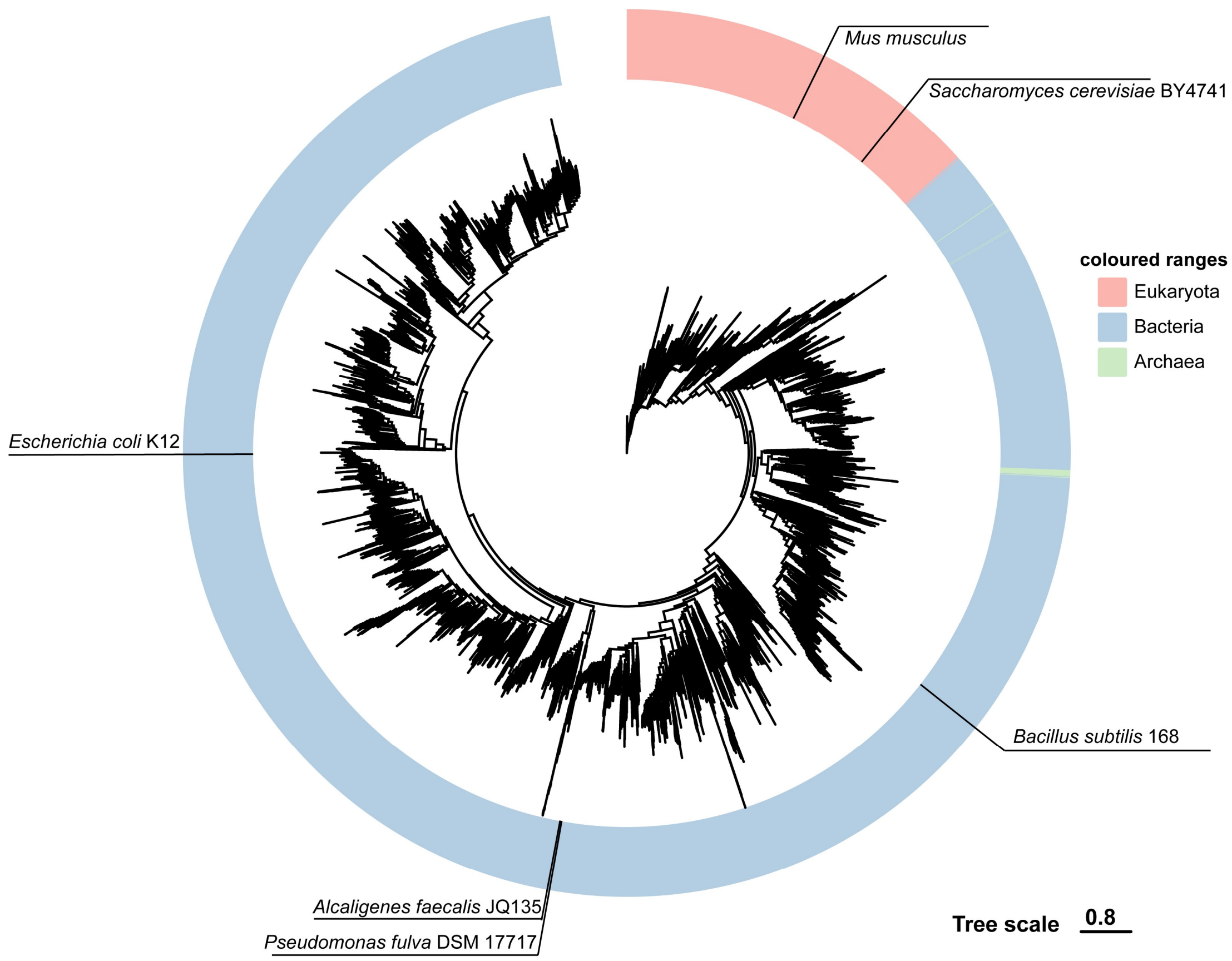

Figure S3 Phylogenetic distribution of YggS homologs across the three domains of life. A circular phylogenetic tree was constructed from 6719 YggS amino acid sequences derived from representative eukaryotic, bacterial and archaeal species. The outer coloured ring denotes taxonomic affiliation: Eukaryota, pink; Bacteria, blue; and Archaea, green. Selected representatives are indicated. The tree highlights a clear separation between eukaryotic and prokaryotic YggS homologs. *Alcaligenes faecalis* JQ135 (WP_316991140.1), *Bacillus subtilis* 168 (WP_088325833.1), *Escherichia coli* K-12 (WP_000997795.1), *Saccharomyces cerevisiae* BY4741 (NP_009517.1), *Pseudomonas fulva* DSM 17717 (WP_023533166.1), *Mus musculus* (NP_473398.1). Scale bar, 0.8 substitutions per site.

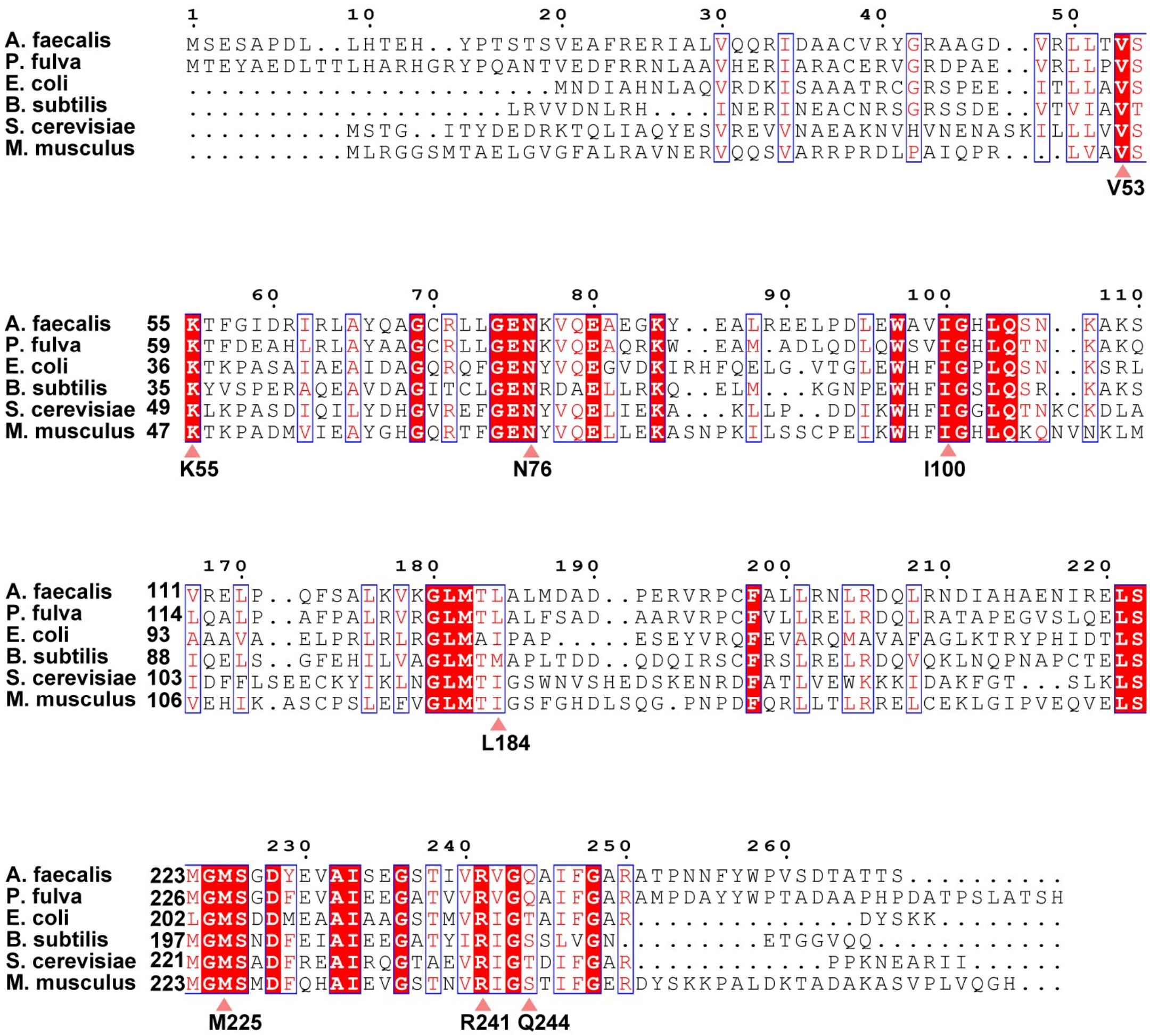

Figure S4 Multiple sequence alignment of YggS homologs and identification of conserved residues. YggS/YggS homologs from *S. cerevisiae: Saccharomyces cerevisiae* BY4741 (NP_009517.1), *M. musculus: Mus musculus* (NP_473398.1), *E. coli: Escherichia coli* K-12 (WP_000997795.1), *A. faecalis* JQ135 YggS (WP_316991140.1), *P. fulva: Pseudomonas fulva* DSM 17717 (WP_023533166.1), and *B. subtilis: Bacillus subtilis* 168 (WP_088325833.1) were aligned. Highly conserved amino acid (V53, K55, N76, I100, L184, M225, R241, and Q244) residues are highlighted in black.

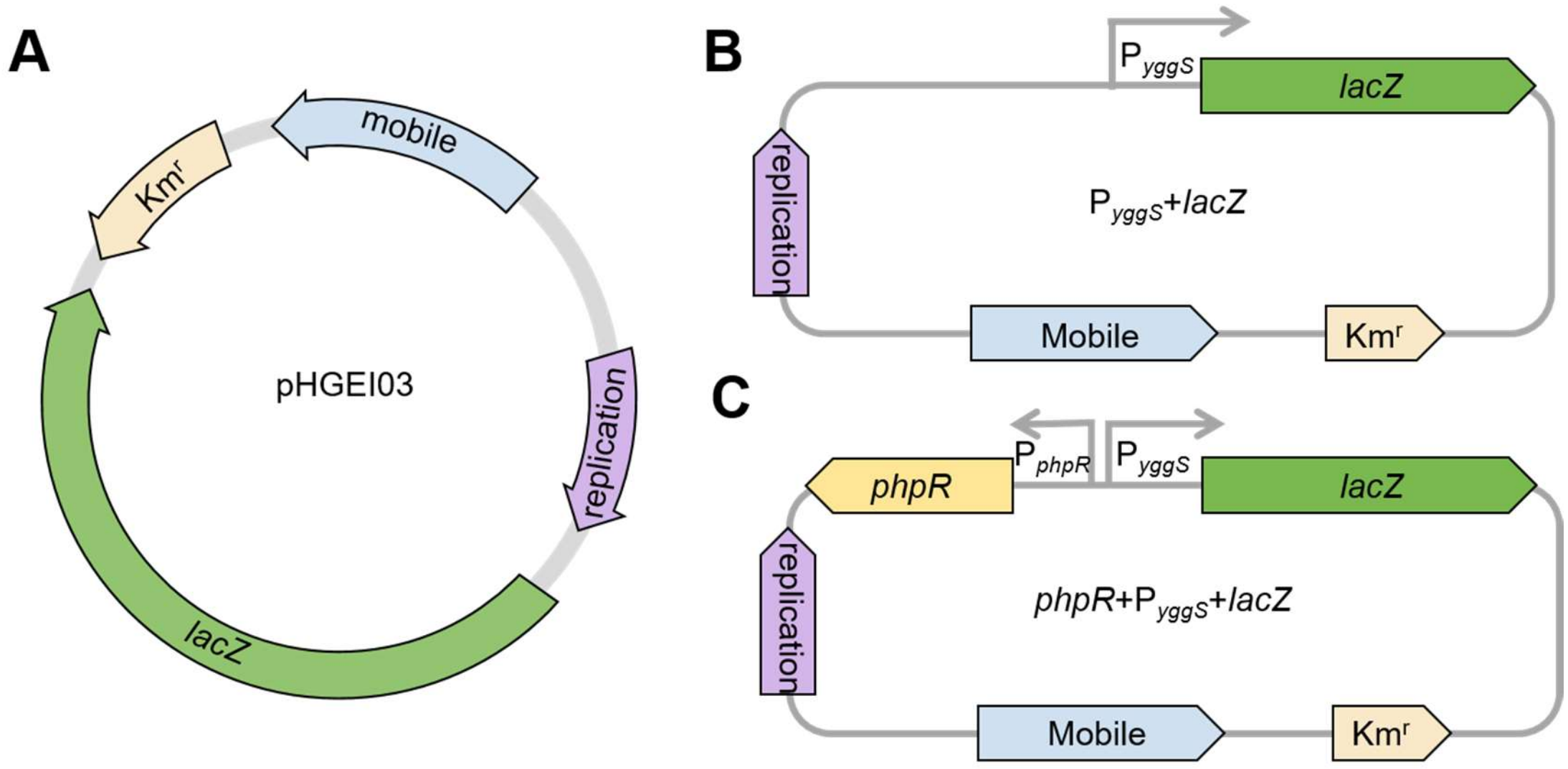

Figure S5 Design of the *lacZ* reporter system for analysis of PhpR-mediated regulation of the *yggS* promoter (Diagram of Figure 3C). (A) Map of the reporter vector pHGEI03, carrying *lacZ*, the kanamycin resistance marker (Km^r), the mobilization region (Mobile), and the replication module. (B) The promoter of *yggS* was cloned upstream of *lacZ* to generate the P_{yggS}-*lacZ* transcriptional reporter. (C) *phpR* was inserted in the orientation corresponding to its native divergent arrangement relative to *yggS, generating the phpR+*P_{yggS}*-lacZ* construct for assessing the effect of PhpR on *yggS* promoter activity. Arrows indicate transcriptional orientation.

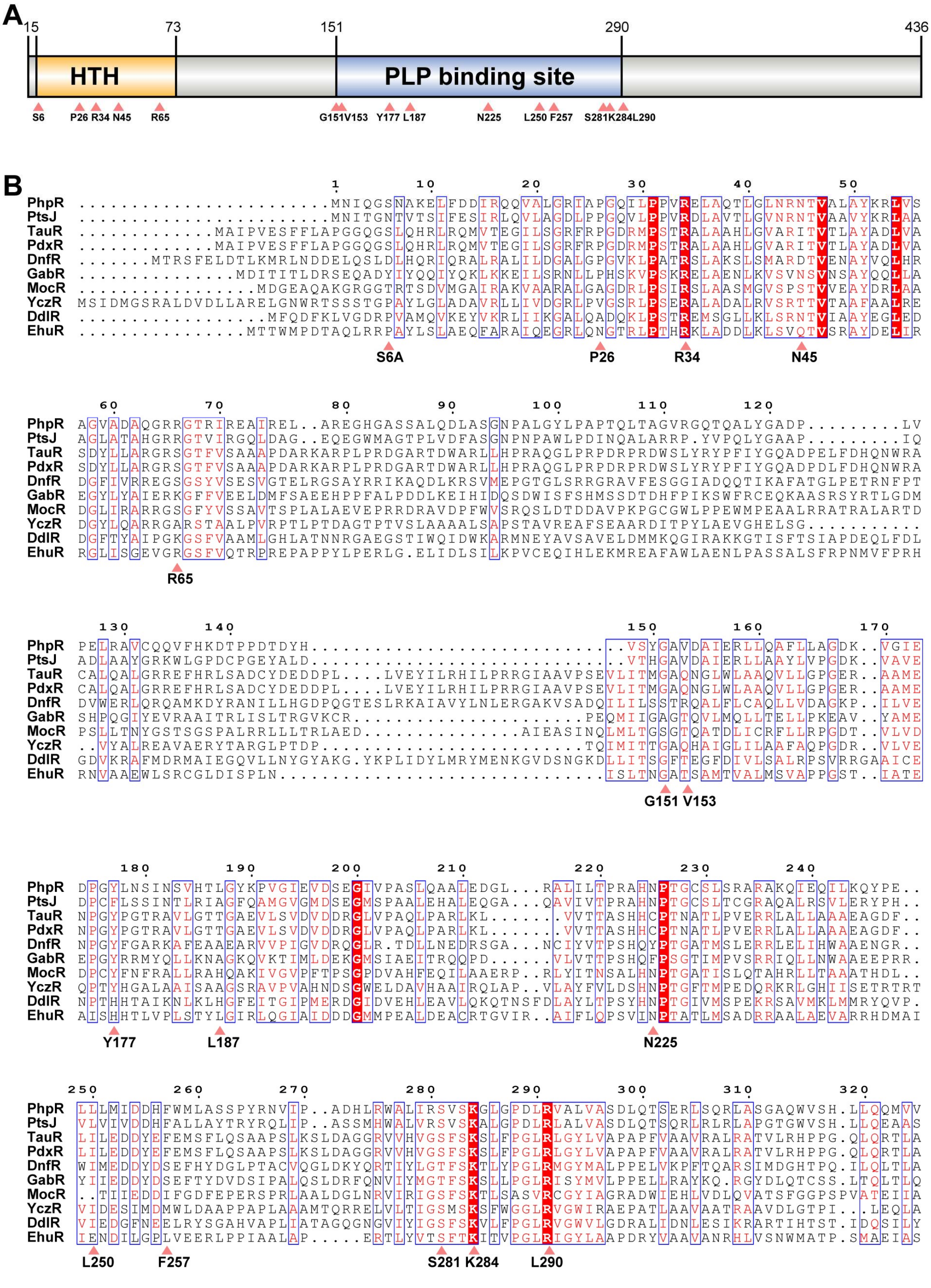

Figure S6 Sequence analysis and conserved residue mapping of PhpR and representative MocR-family regulators. (A) Schematic representation of the domain architecture of PhpR predicted by the NCBI Conserved Domain Database (CDD). The N-terminal winged helix–turn–helix (wHTH) DNA-binding domain and the C-terminal PLP-binding domain are indicated. Conserved amino acid residues selected for mutational analysis are marked below the protein schematic. (B) Multiple sequence alignment of PhpR with representative MocR-family regulators. Amino acid sequence alignment of

PhpR from *A. faecalis* JQ135 with representative MocR-family transcriptional regulators, including PtsJ (WP_130486541.1), TauR (WP_013067948.1), PdxR (WP_013067948.1), DnfR (WP_094197463.1), GabR (WP_095432785.1), MocR (WP_240538014.1), YczR (WP_023985548.1), DdlR (WP_122917329.1) and EhuR (WP_456242959.1). Conserved and similar residues are highlighted. Residues selected for mutational analysis in PhpR are indicated below the alignment, including S6, P26, R34, N45, R65, G151, V153, Y177, L187, N225, L250, F257, S281, K284 and L290. The alignment shows that several residues functionally important for PhpR activity are conserved among MocR-family regulators, supporting their potential roles in DNA recognition and/or effector-dependent regulation.

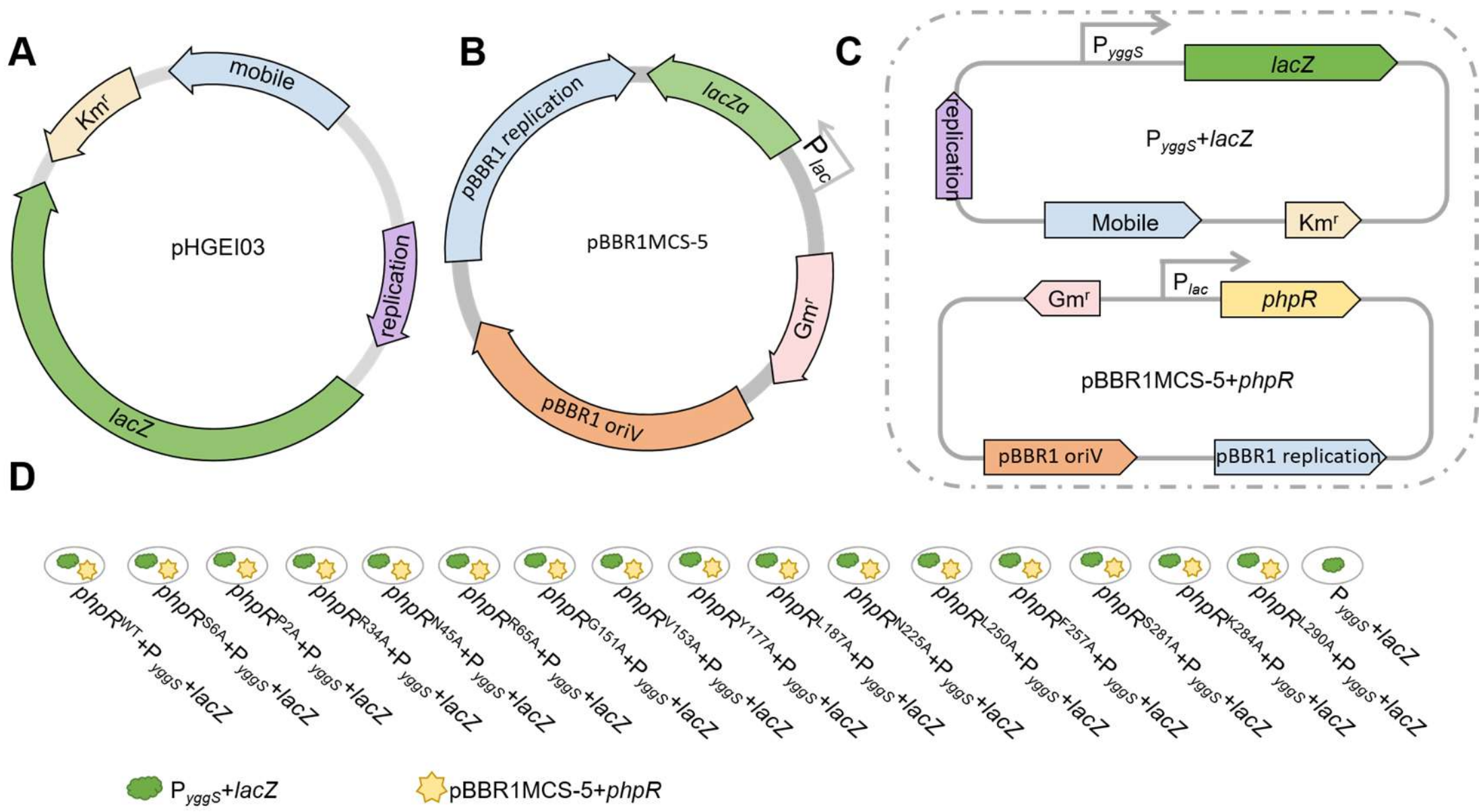

Figure S7 Schematic representation of the plasmid system used for analysis the key residues of PhpR. (A) Map of the reporter vector pHGEI03, carrying *lacZ*, the kanamycin resistance marker (*Km*^{r}), the mobilization region (Mobile), and the replication module. (B) The map of the pBBR1mcs-5, based expression vector carrying *lacZα*, the gentamicin resistance marker (*Gm*^{r}), *pBBR1 oriV* and the replication region. (C) Dual-plasmid system used in Δ*yggS* to examine the amino acid residues required for PhpR-mediated regulation of *yggS* in vivo. The system consisted of the reporter plasmid P_{yggS}-*lacZ*, which was used to measure *yggS* promoter activity, and the expression plasmid pBBR1MCS-5-*phpR*, which was used to express *phpR*. If a PhpR substitution disrupted repression of the *yggS* promoter, *lacZ* activity increased; by contrast, substitutions that did not affect PhpR function caused little or no change in reporter activity. (D) Diagram of the dual-plasmid system used in Figure 5D. The *lacZ* reporter plasmid (P_{yggS}+*lacZ*) and the *phpR* expression plasmid (pBBR1MSC-5+*phpR*) were co-introduced into JQ135 to evaluate the effects of PhpR amino acid residues on the regulation of the *yggS* promoter. Green cells indicate strains carrying only the reporter plasmid (P_{yggS}+*lacZ*), whereas cells marked with yellow stars indicate strains co-harboring the *phpR* expression plasmid (pBBRMSC-5+*phpR*).

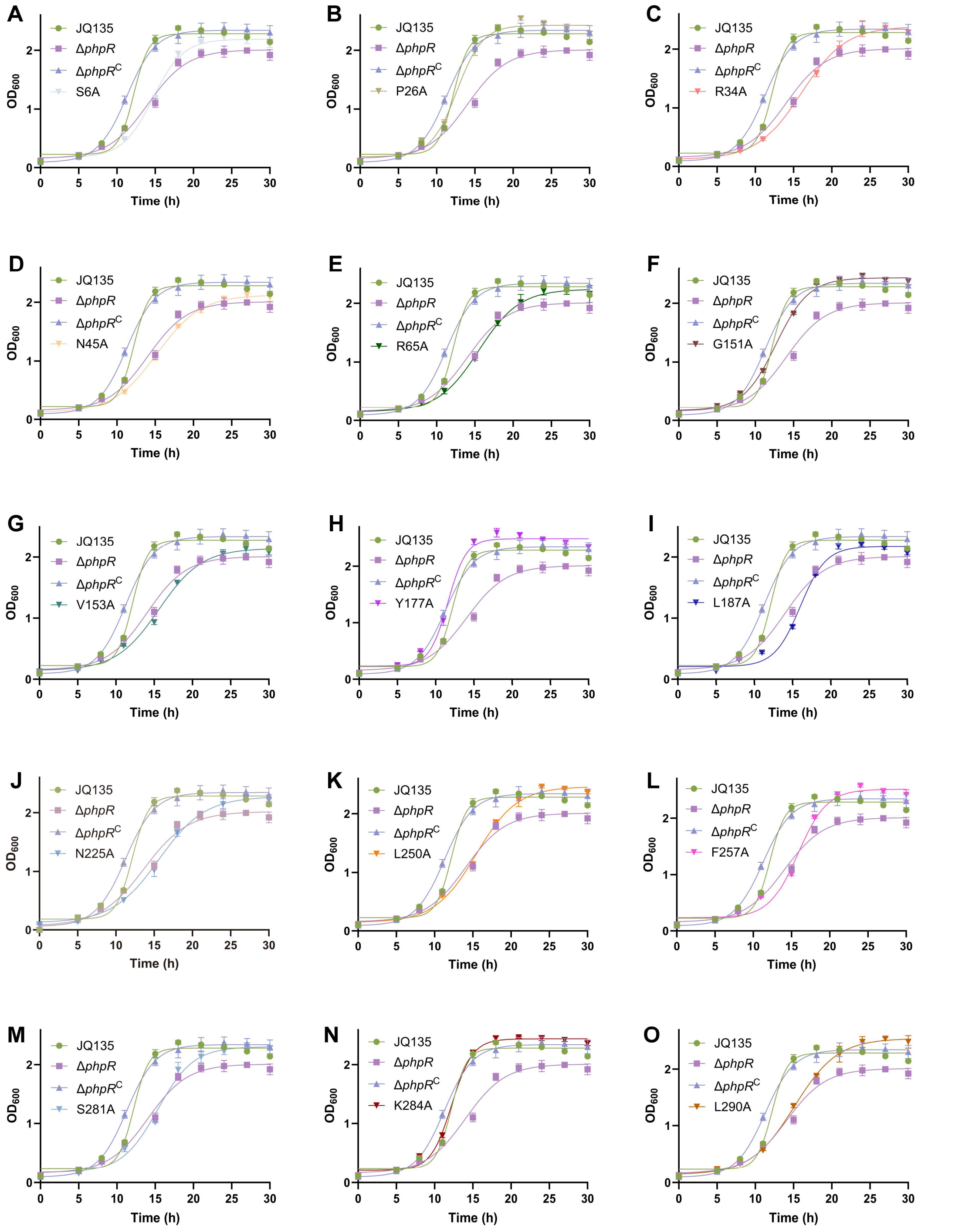

Figure S8 Profiles of growth in JQ135, Δ*phpR* mutant, and the complemented strain Δ*phpR*^{C} in HNM medium. The substituted residues of PhpR analyzed were S6A (A), P26A (B), R34A (C), N45A (D), R65A (E), G151A (F), V153A (G), Y177A (H), L187A (I), N225A (J), L250A (K), F257A (L), S281A (M), K284A (N), and L290A (O). Compared with Δ*phpR*^{C}, several substitutions, including S6A, R34A, N45A, R65A, V153A, L187A, N225A, L250A, F257A, S281A, and L290A, impaired growth to varying extents, whereas P26A, G151A, Y177A, and K284A showed little or no obvious effect. The

growth curves of JQ135, Δ*phpR* and Δ*phpR*^{C} in panels A to O were derived from the same batch culture experiments. All data were obtained simultaneously in a single experiment set but are presented separately for clarity. Data are presented as mean ± SD from three independent experiments.
